# Design and Development of Novel Hybrids Based on Pyrrolo[2,1‐*f*][1,2,4]Triazine and 1‐(Methylpiperidin‐4‐yl) Aniline–Based Analogs: Exploring the Utility as Anticancer Agents via MERTK Inhibition

**DOI:** 10.1111/cbdd.70023

**Published:** 2024-12-05

**Authors:** Balaji Dashrath Sathe, Shivani Jaiswal, Devendra Kumar, Thakur Gurjeet Singh, Nidhi Nainwal, Pramod Rawat, Savita Yadav, Bhupinder Kumar, Ashish Ranjan Dwivedi, S. V. Rathod

**Affiliations:** ^1^ Department of Chemistry Bharatiya Vidya Bhavan College, Chowpatty, Mumbai University Mumbai India; ^2^ Integral Biosciences Pvt Ltd., Drug Discovery Biotech Noida India; ^3^ Institute of Pharmaceutical Research GLA University Mathura India; ^4^ School of Pharmacy Narsee Monjee Institute of Management Studies (NMIMS) Dhule Maharashtra India; ^5^ Centre for Research Impact & Outcome, Chitkara College of Pharmacy Chitkara University Rajpura Punjab India; ^6^ Uttaranchal Institute of Pharmaceutical Sciences Uttaranchal University Dehradun Uttarakhand India; ^7^ Department of Biotechnology Graphic Era (Deemed to Be University) Dehradun India; ^8^ Department of Biotechnology Graphic Era Hill University Dehradun India; ^9^ IES Institute of Pharmacy IES University Bhopal Madhya Pradesh India; ^10^ Department of Pharmaceutical Sciences Chauras Campus, HNB Garhwal University (A Central University) Srinagar Uttarakhand India; ^11^ GITAM School of Pharmacy GITAM (Deemed to Be) University Hyderabad India

**Keywords:** Anticancer, MER tyrosine kinase, Metabolic studies, Sulphonamides, Triazine

## Abstract

Mer‐tyrosine kinase (MERTK), a member of the AXL, TYRO3, and MERTK (TAM) family, is one of the promising targets for cancer treatment. It plays a key role in cancer cell survival and proliferation and regulates immune responses in cancer. The study aimed to rationally design and develop molecules considering the pharmacophoric requirements of MERTK using a multi‐synthetic approach followed by the hybridization of individual pharmacophores. A hybrid drug design approach was employed by hybridization of pyrrolo[2,1‐*f*][1,2,4]triazine and 1‐(methylpiperidin‐4‐yl)aniline pharmacophoric systems to develop novel leads (**1K1–1K5**). The molecules were synthesized via a multi‐step synthetic approach. The synthesized molecules were assessed for their pharmacological potential via cell viability, drug metabolism and pharmacokinetics (DMPK), and MERTK inhibition studies corroborated by in silico studies. **IK5** was found to have an IC_50_ value of 0.36 μM towards A549, followed by 0.42 μM and 0.80 μM against MCF‐7 and MDA‐MB‐231 cells, respectively. Further, the molecules were also analyzed for their microsomal stability and were found to be stable with better intrinsic clearance profiles. The molecules thus pave a strategy for developing novel MERTK inhibitors and their advance in vitro and in vivo assessment in the future.

## Graphical abstract

A hybrid drug design approach was employed to develop novel leads (**1K1–1K5**). The molecules were synthesized via a multi‐step synthetic approach and were assessed for their anticancer potential via cell viability studies, DMPK studies, and MERTK inhibition. Studies were corroborated by in silico studies.

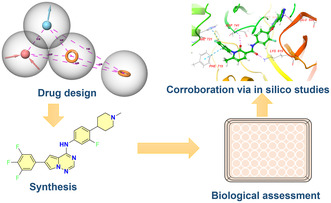

## 1 Introduction

**FIGURE 1:**
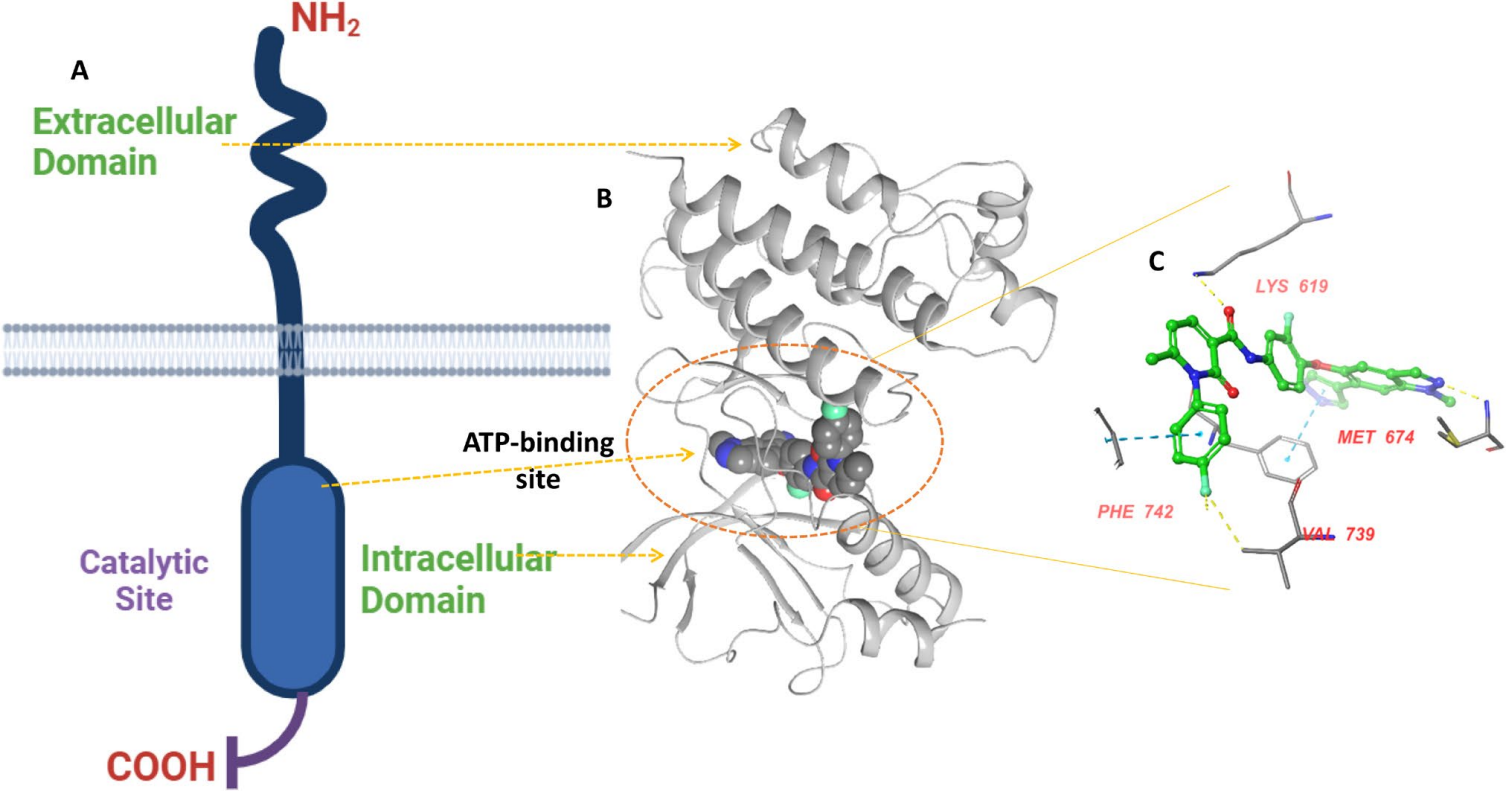
(A) Illustration to portray the MERTK receptor subtype with its three major domains; (B) Ribbon structure of MERTK showing an expanded view of the catalytic site; (C) ATP interaction within the catalytic domain of MERTK.

**FIGURE 2:**
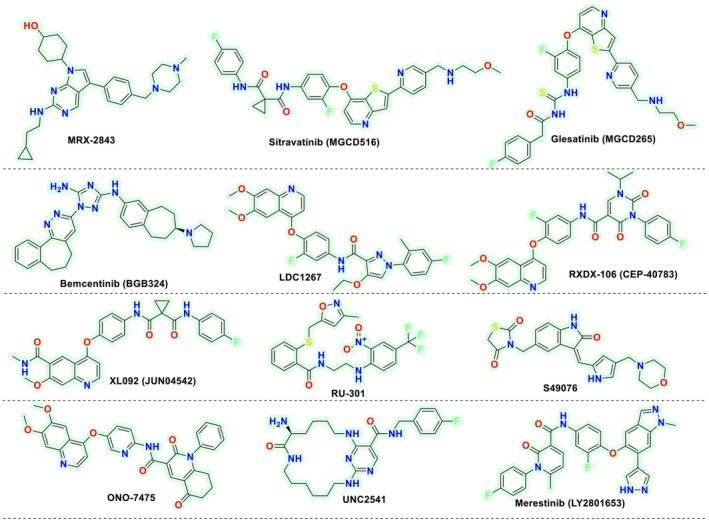
Chemical structures of a few reported MERTK inhibitors in clinical and preclinical trials.

Cancer is a multifaceted disease characterized by uncontrolled cell multiplication and differentiation (Mesnil et al. 1993). This is orchestrated by the numerous intrinsic cellular phenomena not limited to growth factors, overexpression, suppression of apoptotic pathways, elevation in inflammation, and angiogenesis by bypassing cell regulatory checkpoints, allowing the cancer cell survival and prognosis (Finkel, Serrano, and Blasco 2007; Hasan, Pollack, and Cho 2010). Though we have come very far in developing new anticancer agents, we have still failed to control the mortality rate owing to the unavailability of efficacious anticancer agents. The failure is mainly due to drug resistance and toxicity commonly observed with anticancer drug regimens. This, in a sense, is obvious primarily because of the cascade of different receptors and enzymatic networks that orchestrate cancer signaling (Giordano and Petrelli 2008; Wann, Ashley, and Khasraw 2016). Thus, developing inhibitors affecting multiple signaling pathways in cancer is an effective strategy (Amelio et al. 2016). Among numerous targets explored, targeting kinase receptors in cancer treatment is exceedingly beneficial since they play a central role in growth, differentiation, survival, prognosis, and metastasis. The development of specific and selective kinase inhibitors is a part of targeted drug development, which can differentiate cancer cells from normal cells in contrast to traditional chemotherapy. Among different kinases known to be overexpressed in cancer, Mer‐tyrosine kinase (MERTK) belonging to the AXL, TYRO3, and MERTK (TAM) family is an attractive drug target as it is not only involved in cancer cell survival and proliferation but at the same time also regulates the immune responses in cancer (Lahey et al. 2022). Apart from this, MERTK is found to be overexpressed in heterogeneous forms of cancer that include lung cancer, breast cancer, acute myeloid leukemia, and melanoma, among others (Chen and Liu 2021; Davra et al. 2021). Thus, owing to their close linkage with the immune system, targeting MERTK in cancer offers promising therapeutic potential for improving patient outcomes across multiple cancer types (Caetano et al. 2019; Yan et al. 2021). The receptor and druggable site of MERTK (Figure 1) comprises a molecular weight of approximately 110–130 kDa. The size range depends upon the extent of glycosylation. The protein is thought to undergo both homodimerization and heterodimerization, relaying its signaling cascade. The hetero partners of MER include members of the TAM family (AXL and TYRO3). The protein possesses three major domains, including an extracellular domain, allowing it to bind with intrinsic ligands and orchestrate signaling. This is further flanked by the transmembrane domain for anchoring, followed by an intracellular domain. The intracellular domain is the critical site that carries catalytic, i.e., kinase, and regulatory spots. The kinase domain is the catalytic site, as it is the binding site for the ATP. Most small molecules compete with the ATP binding site to mediate their MERTK inhibition. In addition to the catalytic site, MERTK also possesses an allosteric site that may also be targeted since it brings about a conformational change in the protein active site and prevents ATP from binding the catalytic site, thus inhibiting the receptor‐mediated signaling (Cummings et al. 2013; Huelse et al. 2020; Strick and Vollrath 2010). To date, though no drugs have been approved for targeting the MERTK, however, there are a few analogs that have been explored in clinical trials (Figure 2) for their therapeutic efficacy against cancer.

The crucial clinical trial drugs include MRX‐2843, developed by Meryx and presently in Phase 2. The molecule is under trial for treating relapsed or refractory acute myeloid leukemia via inhibition of MERTK. Sitravatinib (MGCD516) is another small molecule developed by Mirati Therapeutics for inhibiting MERTK along with AXL and other kinase targets belonging to the TAM family. The molecule is explored in Phase 2/3 for its utility in non‐small cell lung cancer and renal cell carcinoma. The same pharmaceutical firm has also developed Glesatinib (MGCD265), again under clinical consideration at Phase 1 for MERTK inhibition in solid cancers. Another molecule analyzed for MERTK inhibition is Bemcentinib (BGB324), developed by BerGenBio. The molecule is currently in Phase 2 and is found to target and inhibit MERTK and AXL. It is being explored for its utility in leukemia and solid tumors. Besides this, a few other small molecules, including LDC 1267, RXDX‐106, XL092, RU‐301, S49076, ONO‐7475, UNC2541, and LY2801653, are explored in the preclinical arena for their utility as MERTK inhibitors (Cummings et al. 2015; Koda, Itoh, and Tohda 2018; Lahey et al. 2022; Zhao et al. 2018).

Thus, in the quest to find a putative druggable lead with anticancer asset and MERTK inhibition, we employed a hybrid drug design approach based on pyrrolo[2,1‐*f*][1,2,4]triazine and 1‐(methylpiperidin‐4‐yl)aniline–based pharmacophoric system to develop novel leads (**1K1‐1K5**) (Viegas‐Junior et al. 2007). The triazine ready enjoys the legacy of a privileged nucleus as an anticancer agent due to its potential to modulate various signaling pathways. Over the years, s‐triazine derivatives have significantly progressed with numerous compounds advancing to clinical stages, including PI3K/mTOR and IDH2 inhibitors, attracting considerable interest (Dai et al. 2023). Substitution at the C‐4 and C‐6 positions of the triazine core has augmented anticancer activity by improving selectivity towards target proteins, including BTK and EGFR (Ansari et al. 2018). Triazine derivatives have demonstrated efficacy against solid tumors and hematologic malignancies. Nonetheless, additional optimization is necessary to broaden their applicability, particularly in the context of solid tumors. They can also inhibit critical proteins, including topoisomerases, tyrosine kinases, and cyclin‐dependent kinases, demonstrating their versatility in cancer therapy (Kumar et al. 2019; Sawant, Joshi, and Ansari 2024). The proposed target molecules were rationally developed considering the pharmacophoric requirements of MERTK using a multi‐synthetic approach followed by the hybridization of individual pharmacophores. The final molecules were further assessed using in vitro biological assays, which proved their anticancer potential. Further, the molecules were also analyzed for their microsomal stability and were found to be stable with better intrinsic clearance profiles. The synthetics were also corroborated by in silico techniques to understand their binding and stability within the catalytic site of MERTK. The molecules thus pave a strategy for developing novel MERTK inhibitors and their advance in vitro and in vivo assessment in the future.

## 2 Result and Discussion

### 2.1 Drug Design

**FIGURE 3:**
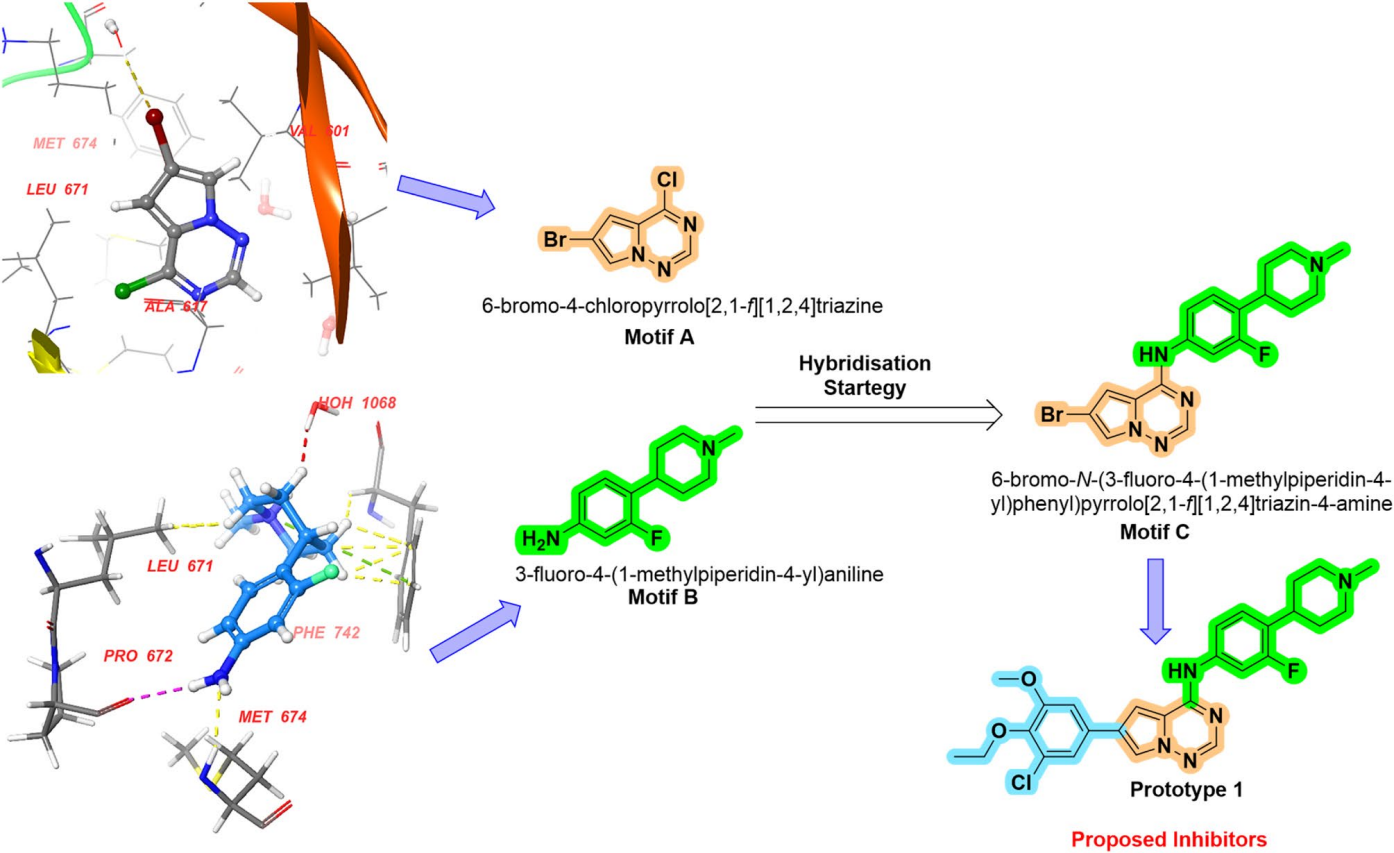
Proposed methodology of drug design for MERTK inhibitors.

An extensive literature survey found that nitrogen‐containing (azaheterocycles) compounds have promising MerTK inhibition (Figure 2). Based on existing literature, we have selected some pharmacophores (fragments) and subjected them to a molecular docking study (Figure 3) using the crystal structure of the MerTK complex with co‐crystallized merestinib (PDB ID: 7AAY) (Pflug et al. 2020). This was done to understand how these pharmacophore moieties fit into the active pocket and interact with specific amino acid residues. The amino acids required for MerTK inhibition include Phe673, Met674, and Arg727 (or Asn728). Among various pharmacophore moieties subjected to docking studies, the 6‐bromo‐4‐chloropyrrolo[2,1‐*f*][1,2,4]triazine (**A**) and 3‐fluoro‐4‐(1‐methylpiperidin‐4‐yl)aniline (**B**) showed the best docking score as well as interaction with amino acids. Motif **A** showed a docking score of −7.9 kcal/mol, and amino acids involved in the interaction are Met674 and Phe742, whereas motif B showed a docking score of −8.5 kcal/mol and amino acids involved in the interaction are Phe742, Leu671, and Pro672, Phe742, and Met674. Consequently, a hybrid drug approach was used to design a new motif, 6‐bromo‐N‐(3‐fluoro‐4‐(1‐methylpiperidin‐4‐yl)phenyl)pyrrolo[2,1‐*f*][1,2,4]triazin‐4‐amine (**C**) by combining these two active pharmacophores through an N‐C linkage. The C was selected as a key precursor, which was further fabricated with different boronic acid derivatives (prototype 1 shown in Figure 3) to develop the present series (1K1‐1K5). Further to explore the active pocket of protein or design new ligands the ligand designer module of Schrodinger software was used.

**FIGURE 4:**
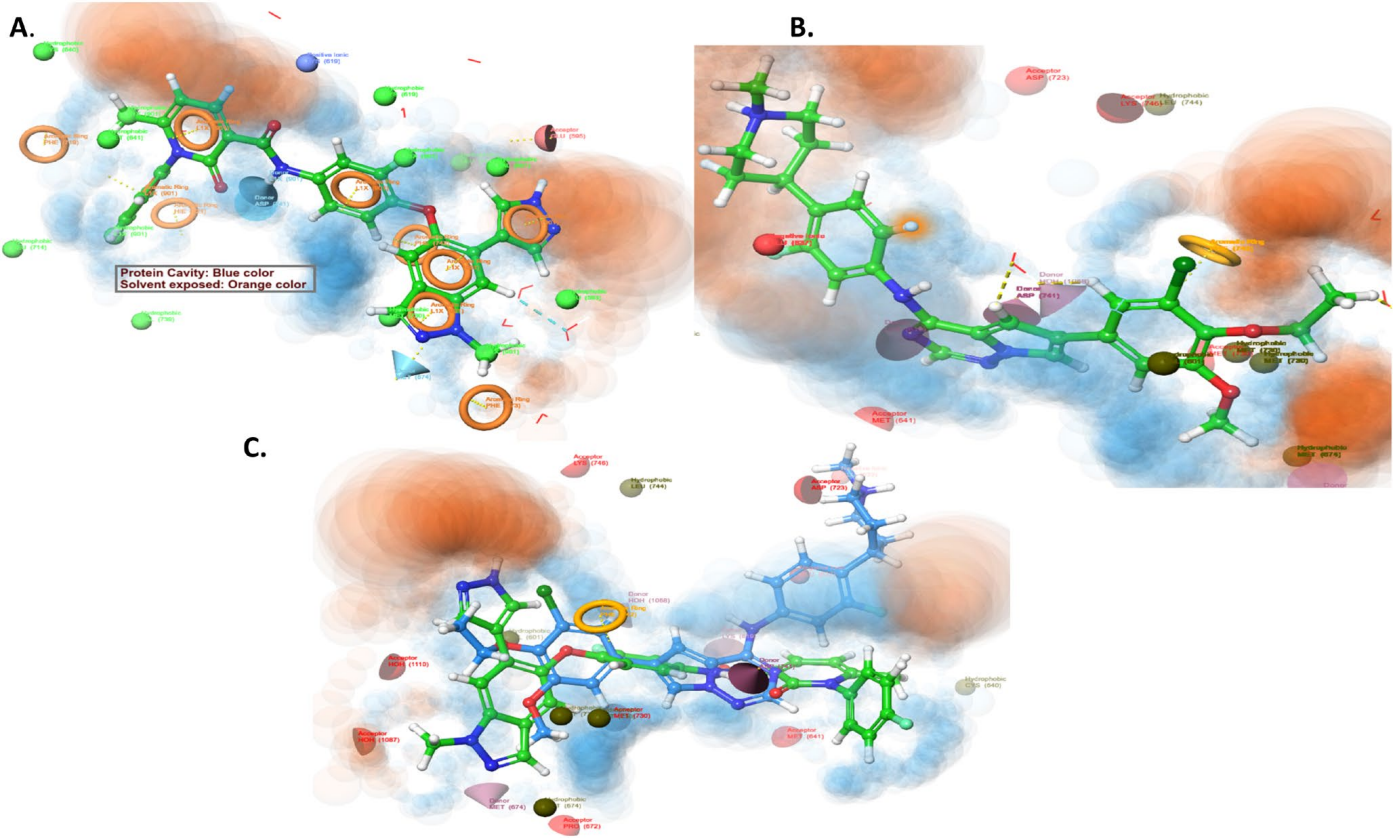
Ligand designer view of the active site; (A) Protein MerTK kinase with reference merestinib; (B) Protein MerTK kinase with prototype 1; (C) Overlaying of prototype1 and merestinib in the active site.

To understand the binding of the proposed molecule (prototype 1) within the active catalytic domain of MERTK (PDB ID: 7AAY), we employed merestinib, a co‐crystallized ligand. The analysis revealed (Figure 4) (protein active cavity (shown in blue) and solvent exposed area (shown in orange)) that both ligand and co‐crystallized ligand occupied a similar binding site. However, a slight pose at the terminal of prototype 1 was seen owing to the bulky addition of boronic acid derivative.

**FIGURE 5:**
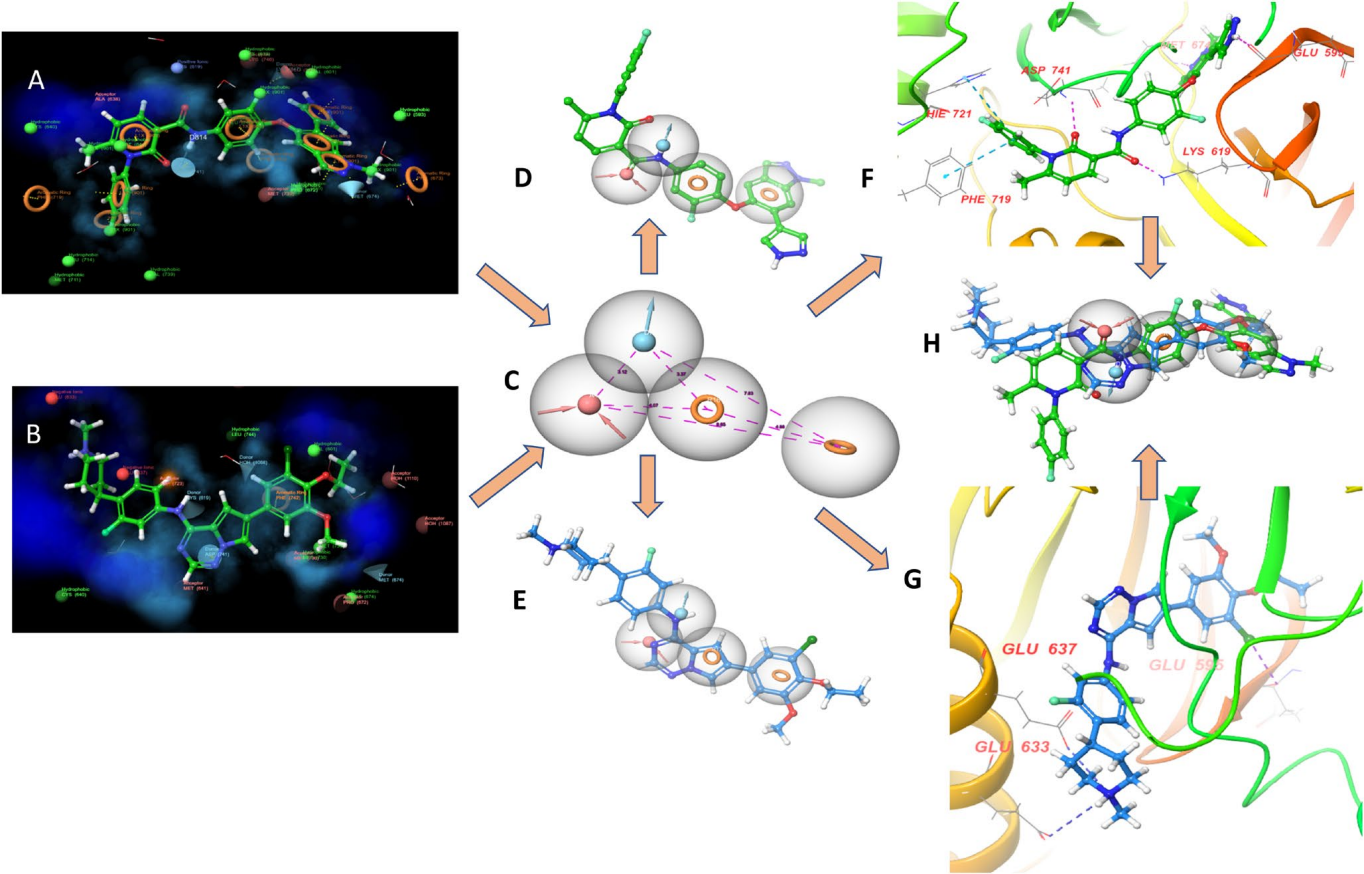
Proposed mapping of prototype 1 (A) and merestinib (B) within the active catalytic site of MERTK to generate the structure‐based pharmacophore model (ADRR) (C). The pharmacophoric model was found to have features alignment with prototype 1 (D) and reference merestinib (E); (F, G) interaction of prototype 1 and merestinib within the active site and (H). Overlaying of prototype 1 and merestinib with pharmacophore is illustrated.

To further validate the findings, we used a conventional approach termed structure‐based pharmacophore modeling (Szwabowski et al. 2023). The receptor–ligand complex was subjected to a structure‐based pharmacophore model in the Phase module of Schrodinger software (Press 2015). To generate the pharmacophore model, PDB ID: 7AAY was used, which resulted in the development of the ADRR model, i.e., four coordinate features possessing one acceptor, one donor, and two ring systems (Figure 5). The pharmacophore model recommended minimum requirements for binding a ligand with the MerTK domain.

### 2.2 Synthetic Methodology

**SCHEME 1:**
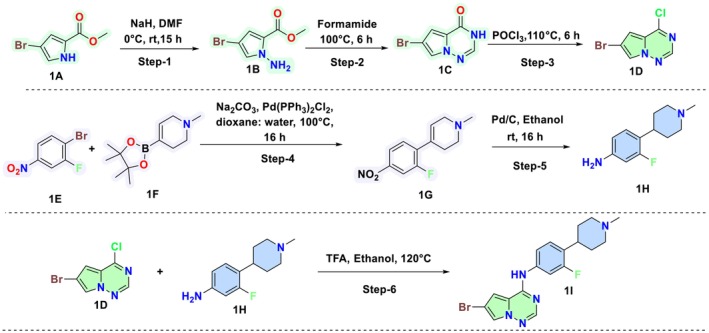
Synthesis of intermediate 6‐bromo‐*N*‐(3‐fluoro‐4‐(1‐methylpiperidin‐4‐yl)phenyl)pyrrolo[2,1‐*f*][1,2,4]triazin‐4‐amine (1I).

**SCHEME 2:**
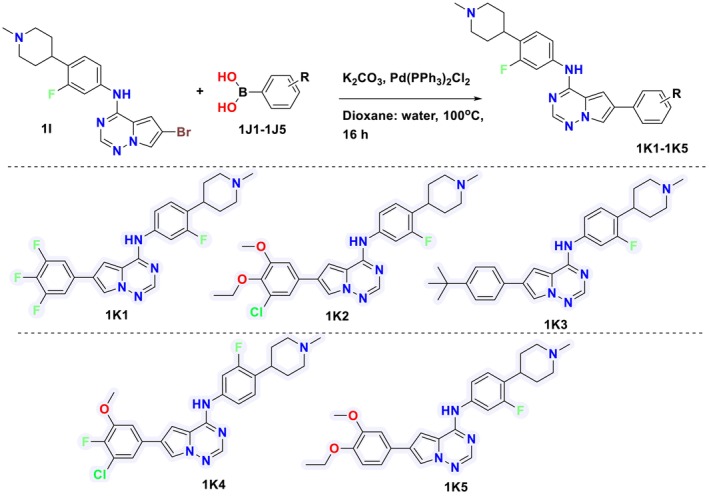
Synthesis of target molecules (**1K1–1K5**).

The target molecules were synthesized through a multi‐step synthetic method, wherein various intermediates were produced as outlined in Synthetic Scheme 1 and subsequently reacted with boronic acid to yield the final target compound **1K1‐1K5** (Synthetic Scheme 2). Briefly, methyl 4‐bromo‐1H‐pyrrole‐2‐carboxylate (**1A**, 5.99 g, 21.00 mmol) in dry DMF (200 mL) was reacted with sodium hydride (NaH, 60% dispersion in mineral oil, 1.01 g, 25.2 mmol) followed by the addition of aminooxy‐diphenylphosphine oxide (5.87 g, 25.19 mmol) to give methyl 1‐amino‐4‐bromo‐1H‐pyrrole‐2‐carboxylate (**1B**, 4.71 g, 74.5%) which was subjected to react with formamide (3 mL) at 100°C to give 6‐bromopyrrolo[2,1‐*f*][1,2,4]triazin‐4(3H)‐one (**1C**). **1C (**100 mg, 2.8 mmol) was reacted with phosphorous oxychloride (POCl_3_, 5 mL) at 110°C to give intermediate 6‐bromo‐4‐chloropyrrolo[2,1‐*f*][1,2,4]triazine (**1D**, 100 mg, 90% yield). On the other hand, intermediate **IH** was synthesized from starting material 1‐bromo‐2‐fluoro‐4‐nitrobenzene (**1E**, 3 g, 13.76 mmol) which on reaction with 1‐methyl‐4‐(4,4,5,5‐tetramethyl‐1,3,2‐dioxaborolan‐2‐yl)‐1,2,3,6‐tetrahydropyridine (**1F**, 5.16 g, 23.13 mmol) and sodium carbonate (3.2 g, 30.66 mmol) in the presence of Pd(PPh_3_)_2_Cl_2_ (0.533 g, 0.766 mmol) yielded 4‐(2‐fluoro‐4‐nitrophenyl)‐1‐methyl‐1,2,3,6‐tetrahydropyridine (**1G**, 3 g, 91%). The nitro group of **IG** (2.33 g, 7.23 mmol) was converted to an amine via palladium‐catalyzed (0.433 g) reduction to yield intermediate 3‐fluoro‐4‐(1‐methylpiperidin‐4‐yl)aniline (**1H**, 1.9 g, 82%). In the next step, intermediates **1D** (1.0 g, 4.34 mmol) and **1H** (0.902 g, 4.34 mmol) were condensed in the presence of TFA (2 mL) and ethanol (15 mL) to give the next intermediate 6‐bromo‐N‐(3‐fluoro‐4‐(1‐methylpiperidin‐4‐yl)phenyl)pyrrolo[2,1‐*f*][1,2,4]triazin‐4‐amine (**1I**, 800 mg, 48%). In Synthetic Scheme 2, the intermediate **1I** (100 mg, 0.2473 mmol) in the presence of potassium carbonate (K_2_CO_3_, 102 mg, 0.7419 mmol) and Pd(PPh_3_)_2_Cl_2_ (10 mg, 0.012 mmol) was reacted with various substituted boronic acids (**1J1‐1J5**, 0.4946 mmol) to yield the final products **1K1‐1K5** in good yields. Thin‐layer chromatography (TLC) and liquid chromatography–mass spectrometry (LCMS) were employed to determine the reaction progress continually at each step. As required, the final products were purified using flash or column chromatography (5%MeOH/DCM). The final structural elucidation of intermediates and final products was performed using ^1^H NMR, ^13^C NMR, and LCMS spectral analysis.

### 2.3 Biological Assessment

To investigate whether the synthetic molecules have the anticancer potential and can inhibit the MER TK, we performed a series of biological experiments.

#### 2.3.1 Cytotoxicity Studies

To decipher whether the synthetic molecules possess anticancer potential, we assessed them for their cytotoxicity in three cell lines. This includes lung cancer cells (A549) and breast cancer cells (MCF‐7 and MDA‐MB‐231). The analysis revealed (Table 1) compound **1K5** to possess excellent cytotoxicity towards each cancer cell line and possessed nanomolar range IC50 values. **IK5** was found to have an IC_50_ value of 0.36 μM towards A549, followed by 0.42 μM and 0.80 μM, respectively, in MCF‐7 and MDA‐MB‐231 cells. Compound **1K2** was also found to portray a nanomolar range inhibition towards MCF‐7 cells with IC_50_ of 0.44 μM, while **IK4** exhibited IC_50_ of 0.89 μM. The remaining compounds were also found to portray the inhibition at a low micromolar range and better than the cisplatin, employed as a positive control during the experiment. Further, the synthetics were found to have selective anticancer potential as they do not offer any toxicity towards normal HPBMCs employed.

**TABLE 1:** IC_50_ values of target compounds **IK1**–**IK5** against different cancer cell lines.

Entry name	IC_50_ values (μM) (mean ± SD)		
	A549	MCF‐7	MDA‐MB‐231
**1K1**	8.63 ± 0.021	1.28 ± 0.014	4.02 ± 0.015
**1K2**	1.18 ± 0.019	0.44 ± 0.015	1.70 ± 0.011
**1K3**	1.44 ± 0.017	1.42 ± 0.021	4.06 ± 0.019
**1K4**	0.89 ± 0.011	1.14 ± 0.011	1.47 ± 0.014
**1K5**	0.36 ± 0.019	0.42 ± 0.017	0.80 ± 0.011
**Cisplatin**	5.86 ± 0.009	4.93 ± 0.13	5.42 ± 0.09

#### 2.3.2 DMPK Studies

To understand the intrinsic pharmacokinetics of synthetics and their stability in relation to human liver microsomal (HLM) and mouse liver microsomal (MLM), we performed the DMPK studies. The analysis revealed (Table 2) that **1K5** possesses the maximum stability with a T1/2 of 396.70 min in HLM. In contrast, **IK3** was found to show poor stability with half‐lives of 49.40 min and 36.90 min, respectively, in MLM and HLM. The half‐lives for the remaining compounds were found to be aligned with the MLM data. However, in contrast to this, 1K4 possessed better stability in MLM (238.40 min) than the HLM (75.20 min).

**TABLE 2:** DMPK profile of target compounds **1K1–1K5**.

Entry name	HLM	MLM	*T*_1/2_ (HLM)	*T*_1/2_ (MLM)	CLint^a^ (HLM)	CLint^a^ (MLM)
**1K1**	71.67	70.02	62.40	58.30	22	24
**1K2**	82.75	83.36	109.80	114.20	13	12
**1K3**	65.65	56.95	49.40	36.90	28	38
**1K4**	75.84	91.65	75.20	238.40	18	6
**1K5**	94.89	79.65	396.70	92.40	3	15

*Note:* Units: HLM: μL/min/mg protein; MLM: μL/min/mg protein; *T*_1/2_ (HLM): min; *T*_1/2_ (MLM): min; CLint (HLM): μL/min/mg protein; CLint (MLM): μL/min/mg protein.

^**a**^ CLint is one of the pharmacokinetic parameters related to liver effectiveness in drug metabolism.

Considering the intrinsic clearance, **1K3** was found to have the highest clearance, indicating a faster metabolization by microsomal enzymes. The **IK5** showed the lowest intrinsic clearance in HLM (3 μL/min/mg), indicating slow metabolism, which aligns well with its t_1/2_. The results are important since they are critical parameters to judge the pharmacokinetics of the prosed compounds and their utility to be safe and efficacious future drug candidates.

#### 2.3.3 MERTK Inhibition

All the synthesized molecules were assessed for their kinase inhibitory activity against MERTK using a kinase inhibition assay. The results demonstrated a weak inhibitory potential of the synthesized compounds against MERTK. One of the compounds, **IK5**, displayed approx. 42% inhibition of MERTK, whereas all other compounds exhibited suboptimal inhibition at 1 and 10 μM test concentrations. IK5 exhibited comparable inhibition to positive control staurosporine.

**FIGURE 6:**
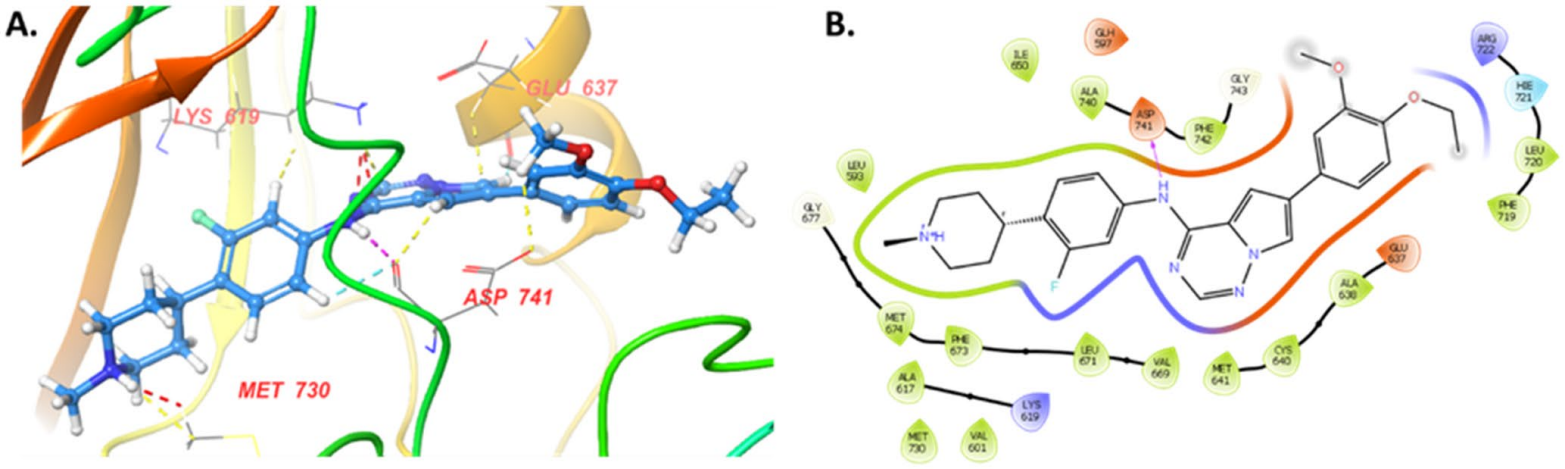
Interaction (A. 2D; B. 3D) of investigational compound **IK5** within the active domain of MERTK (PDB ID: 7AAY).

To understand the binding pose of the most potent compound, **1K5**, we performed molecular docking studies. Docking study of hit compound carried with protein MerTK complexed with reference merestinib (PDB ID: 7AAY) using Maestro Schrodinger suite. The analysis revealed **(**Figure 6 **) that** compound **IK5** has a docking score of −9.1 kcal/mol and possesses hydrogen bond interactions with amino acids Met730, ASP741, LYS679, and GLU737 which provides the stability to complex and were found to remain conserved as laid in the drug design section.

**FIGURE 7:**
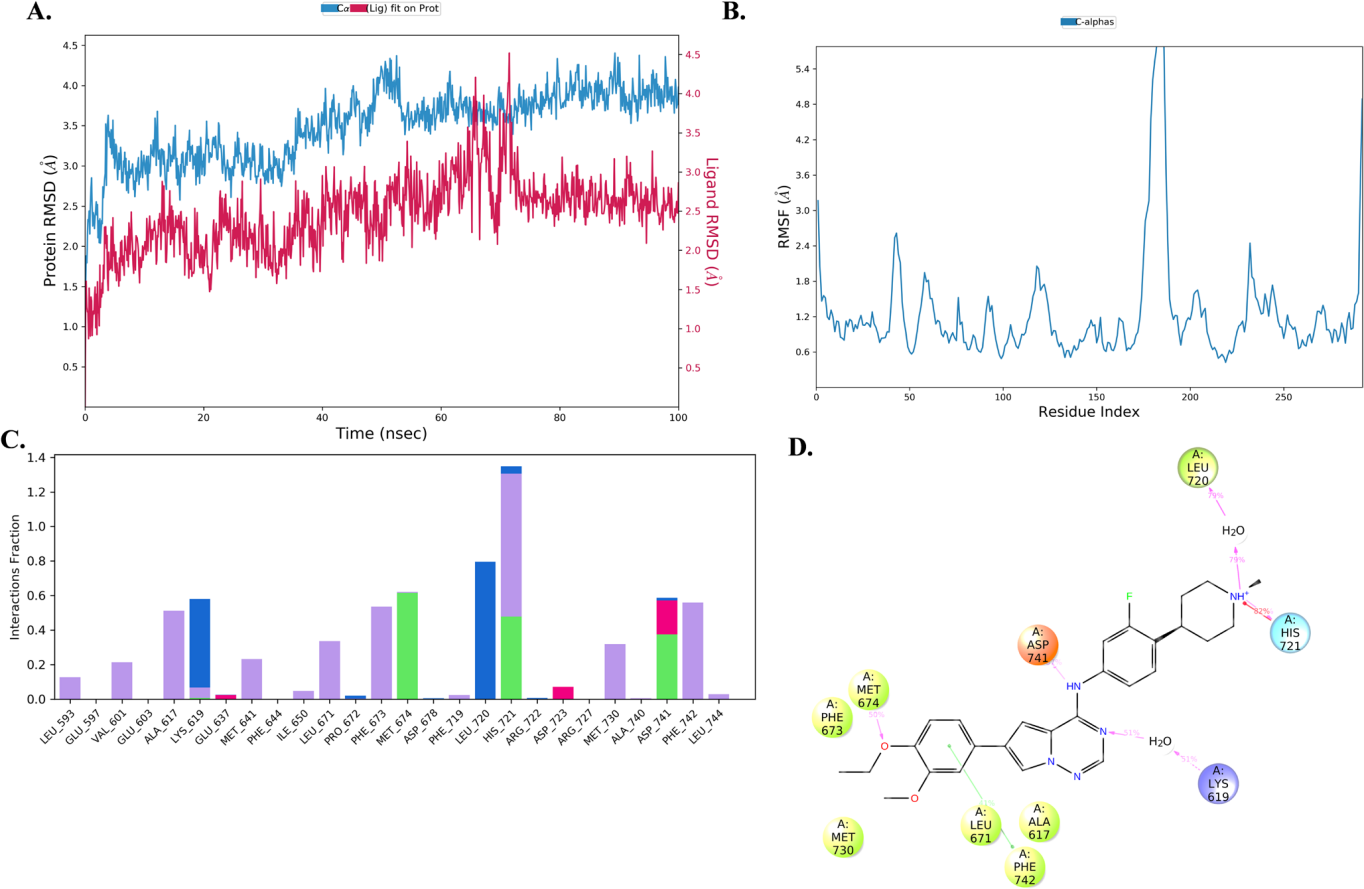
(A) PL‐RMSD of IK5 with protein MerTK complex (PDB ID: 7AAY). (B) RMSF of the MerTK protein. (C) PL‐contacts histogram E. (D) Interaction diagram of ligand with amino acids.

Further, to understand the stability of the protein–ligand complex, we performed the MD analysis of the compound **IK5** with protein MerTK complex (PDB ID: 7AAY). The complex was subjected to molecular dynamics stimulation for 100 ns. Analysis of the root‐mean‐square deviation (RMSD) graph indicated that the protein remained stable during the 100 ns simulation, exhibiting minimal fluctuations between 2.5 and 4.5 Å, as illustrated in (Figure 1A). Next, the ligand RMSD graph analyses where the ligand showed slight fluctuation around 75 ns; after that, it remained stable over the 100 ns simulation time within the 1.5–2.5 Å (Figure 7A). Thus, the range of ligand RMSD indicated that ligands remain in the active site of the proteins. The protein root‐mean‐square fluctuation (RMSF) graph indicated that fluctuation was observed between the 170–200 (5.4 Å) amino acid residues depicted in Figure 7B. The protein–ligand (PL) contact histogram indicated that ASP 741 (57%) and MET 674 were involved in the hydrogen bonding and Phe742 (41%) was involved in the pi–pi stacking interactions (Figure 7C). This amino acid Phe673, Leu720, and His721 also aid the stability of PL complex Figure 7D.

#### 2.3.4 ADMET Prediction

Evaluating the pharmacokinetic features and toxicity profile of new molecules is a paramount task to prevent failure in clinical studies. Thus, PreADME and ProTox (Freely available online web‐based prediction tools) were employed to evaluate the ADME and toxicity profile of these newly designed molecules, and the results for same are depicted in Table 3. All the designed molecules lie in the class 4 category of toxicity profile, indicating that below 1000 mg/kg dose, these molecules are safer for further studies. The molecules lie well within the range of parameters as per Lipinski's rule of five. The molecular weight, rotatable bonds, hydrogen bond donors, and acceptors are acceptable for the development as drug candidates. The molecules cannot cross the blood–brain barrier which further attenuates the chances of neurotoxicity on administration. The safer and non‐toxic behavior of these molecules suggests them as potential pharmacophores for designing and developing safer anticancer compounds targeting MERTK.

**TABLE 3:** Physicochemical properties of **1K1–1K5** predicted in silico.

S. No.	Compound ID	MW	LogP o/w	HB donor	HB acceptor	BBB	TPSA (Å)	ROF	Protox Predicted LD_50_ (mg/kg)
1.	**1K1**	455.45	5.57	1	7	No	45.46	4	1000 mg/kg Class 4
2.	**1K2**	510.00	4.46	1	6	No	63.92	7	1000 mg/kg Class 4
3.	**1K3**	457.59	5.27	1	4	No	45.46	5	1000 mg/kg Class 4
4.	**1K4**	483.94	4.96	1	6	No	54.69	5	1000 mg/kg Class 4
5.	**1K5**	475.56	4.00	1	6	No	63.92	7	1000 mg/kg Class 4

Abbreviations: BBB, Blood–brain barrier permeability; HB, hydrogen bond; LogP, oil–water partition coefficient; ROF, Lipinski's Rule of Five; TPSA, topological polar surface area.

## 3 Conclusion

Cancer is a multifaceted disease orchestrated by numerous intrinsic cellular phenomena not limited to growth factors, overexpression, suppression of apoptotic pathways, elevation in inflammation, and angiogenesis by bypassing cell regulatory checkpoints, allowing the cancer cell survival and prognosis. Besides exhaustive efforts, we have still failed to control the mortality rate owing to the unavailability of efficacious anticancer agents. Drug resistance and toxicity of drug molecules are major hurdles in anticancer drug development. Among numerous targets explored, targeting kinase receptors in cancer treatment is exceedingly beneficial since they play a central role in growth, differentiation, survival, prognosis, and metastasis. A MERTK, a member of the TAM family, is one of the promising targets for cancer treatment among kinases as it plays a key role in cancer cell survival and proliferation along with the regulation of immune responses in cancer. In a quest to develop novel MERTK inhibitors, a hybrid drug design approach was employed by hybridization of pyrrolo[2,1‐*f*][1,2,4]triazine and 1‐(methylpiperidin‐4‐yl)aniline pharmacophoric systems to develop novel leads (**1K1‐1K5**).

The synthesized compounds were evaluated for their cytotoxicity against A549, MCF‐7, and MDA‐MB‐231 cancerous cell lines. In cytotoxicity studies, **IK5** was found to have an IC_50_ value of 0.36 μM towards A549, followed by 0.42 μM and 0.80 μM against MCF‐7 and MDA‐MB‐231 cells, respectively. Compound **1K2** was also found to portray a sub‐micromolar range inhibition towards MCF‐7 cells with IC_50_ of 0.44 μM, while **IK4** exhibited IC_50_ of 0.89 μM against A549. Further, the molecules were also analyzed for their microsomal stability and were found to be stable with better intrinsic clearance profiles. In MERTK inhibitory studies, **IK5** exhibited comparable inhibitory potential to the positive control drug staurosporine. The synthetics were also corroborated by in silico techniques to understand their binding and stability within the catalytic site of MERTK. The molecular docking studies confirmed the suitable binding interactions at the active site of MERTK and pharmacokinetic prediction studies confirmed the appropriate ADME profile of structural features of designed molecules. The molecules thus pave a strategy for developing novel MERTK inhibitors and their advance in vitro and in vivo assessment in the future.

## 4 Material and Methods

### 4.1 Synthesis and Characterization of Compounds

#### 4.1.1 Synthesis of Methyl 1‐Amino‐4‐Bromo‐1H‐Pyrrole‐2‐Carboxylate (1B)

The solution of methyl 4‐bromo‐1H‐pyrrole‐2‐carboxylate (**1A**, 5.99 g, 21.00 mmol) in dry DMF (200 mL) was stirred at 0°C and sodium hydride was added (60% dispersion in mineral oil, 1.01 g, 25.2 mmol) at 0°C. The reaction mixture was brought to room temperature and stirred for 15 min followed by the addition of aminooxy‐diphenylphosphine oxide (5.87 g, 25.19 mmol) and left for stirring for a further 15 h. The reaction progress was determined using TLC (60% ethyl acetate in hexane). After confirmation of complete consumption of the reactant, ice‐cold water (100 mL) was added to the reaction mixture, followed by extraction using ethyl acetate (100 mL X 2). After separating the organic layer, an aqueous portion was again extracted with ethyl acetate (100 mL X 2), and the combined organic layer was dried over anhydrous Na_2_SO_4_. The dried organic solution was concentrated under a vacuum using a rotary evaporator to obtain the crude product triturated with dichloromethane‐hexanes (1:1 v/v, 10 mL). The remaining solid was washed with hexane (100 mL) and dried under vacuum to yield the desired product methyl 1‐amino‐4‐bromo‐1H‐pyrrole‐2‐carboxylate (**1B**, 4.71 g, 74.5%).

#### 4.1.2 Synthesis of 6‐Bromopyrrolo[2,1‐*f*][1,2,4]Triazin‐4(3H)‐one (1C)

In the second step, the solution of **1B** (0.966 g, 4.4 mmol) in formamide (3 mL) was subjected to heating along with stirring at 100°C for 6 h without any additional solvent. The reaction progress was determined using TLC. On completion of the reaction, ice‐cold water (30 mL) was added to the reaction mixture, followed by extraction using ethyl acetate (50 mL X 2), washing with water (50 mL X 5), and brine solution (100 mL X 3). The combined organic layer was dried over anhydrous Na_2_SO_4_. The dried organic solution was concentrated under a vacuum using a rotary evaporator to obtain the desired crude product, which was triturated ethyl acetate and hexanes (4:1, 30 mL) to give 6‐bromo‐3H‐pyrrolo[2,l‐*f*][l,2, 4]triazin‐4‐one (**1C**, 0.61 g, 65%).

#### 4.1.3 Synthesis of 6‐Bromo‐4‐Chloropyrrolo[2,1‐*f*][1,2,4]Triazine (1D)

The solution of **IC** (100 mg, 2.8 mmol) in phosphorus oxychloride (5 mL, as a source of chlorination and solvent) was subjected to stirring at room temperature followed by heating at 110°C for 5 h. The reaction progress was determined using TLC. After confirmation of complete consumption of the reactant, it was brought to room temperature and concentrated under reduced pressure. The ice‐cold water (50 mL) was added to the concentrated reaction mixture, followed by extraction with dichloromethane (20 mL X 3). The organic layer was dried over anhydrous Na_2_SO_4_. The dried organic solution was concentrated under vacuum using a rotary evaporator to give 6‐bromo‐4‐chloropyrrolo[2,1‐*f*][1,2,4]triazine (**1D**, 100 mg, 90% yield) as a brown solid.

#### 4.1.4 Synthesis of 4‐(2‐Fluoro‐4‐Nitrophenyl)‐1‐Methyl‐1,2,3,6‐Tetrahydropyridine (IG)

The 1‐bromo‐2‐fluoro‐4‐nitrobenzene (**1E**, 3 g, 13.76 mmol) was dissolved in dioxane (20 mL) and water (10 mL) followed by addition of 1‐methyl‐4‐(4,4,5,5‐tetramethyl‐1,3,2‐dioxaborolan‐2‐yl)‐1,2,3,6‐tetrahydropyridine (**1F**, 5.16 g, 23.13 mmol) and sodium carbonate (3.2 g, 30.66 mmol). The nitrogen gas was purged from the mixture to remove all air and Pd(PPh_3_)_2_Cl_2_ (0.533 g, 0.766 mmol) and left stirring at 95°C for 16 h. The reaction progress was determined using TLC (30% ethyl acetate: hexane). After confirmation of complete consumption of the reactant, the solvent was evaporated under reduced pressure. The water (200 mL) was added to the concentrated reaction mixture followed by extraction with ethyl acetate (200 mL). The organic layer was washed with brine solution (200 mL x 3), dried over anhydrous Na_2_SO_4_, and concentrated under reduced pressure to yield a crude product. This crude product was further purified using Combi‐Flash to afford 4‐(2‐fluoro‐4‐nitrophenyl)‐1‐methyl‐1,2,3,6‐tetrahydropyridine (**1G**, 3 g, 91%) as off‐white solid for further reaction.

#### 4.1.5 Synthesis of 3‐Fluoro‐4‐(1‐Methylpiperidin‐4‐yl)aniline (IH)

The solution of 4‐(2‐fluoro‐4‐nitrophenyl)‐1‐methyl‐1,2,3,6‐tetrahydropyridine (**1G**, 2.33 g, 7.23 mmol) in ethanol (20 mL) was stirred at room temperature followed by addition of 10% Pd/C (0.433 g) and left for stirring for further 20 h under hydrogen atmosphere. The reaction progress was determined using TLC (5% MeOH: DCM). After confirmation of complete consumption of the reactant, the reaction mixture was filtered through a celite bed, followed by washing with ethanol (20 mL × 2). The collected filtrate was subjected to reduced pressure to yield 3‐fluoro‐4‐(1‐methylpiperidin‐4‐yl)aniline (**1H**, 1.9 g, 82%) as a solid.

#### 4.1.6 Synthesis of 6‐Bromo‐N‐(3‐Fluoro‐4‐(1‐Methylpiperidin‐4‐yl)phenyl)pyrrolo[2,1‐*f*][1,2,4]Triazin‐4‐Amine (1I)

TFA (2 mL) and 3‐fluoro‐4‐(1‐methylpiperidin‐4‐yl)aniline (**1H**, 0.902 g, 4.34 mmol) were added to the ethanolic (15 mL) solution of 6‐bromo‐4‐chloropyrrole[1,2,‐f][1,2,4]triazine (1D, 1.0 g, 4.34 mmol) kept for stirring at room temperature. The resulting mixture was overnight heated at 120°C. TLC and LCMS were employed to determine the reaction progress for the synthesis of this intermediate. After confirmation of complete consumption of reactants, the solvent was evaporated under reduced pressure to yield the crude product which was further triturated with isopropyl alcohol and hexane to obtain yield 6‐bromo‐N‐(3‐fluoro‐4‐(1‐methylpiperidin‐4‐yl)phenyl)pyrrolo[2,1‐*f*][1,2,4]triazin‐4‐amine (**1I**) as a desired product (800 mg, 48%). The product was further characterized using LCMS and ^1^H NMR.

^1^H NMR (400 MHz, DMSO‐*d*_6_) δ ppm 9.95 (s, 1,H), 8.09 (s, 1H), 8.01 (d, J = 1.43 Hz, 1H), 7.86 (d, J = 14.31 Hz, 1H), 7.52 (d, J = 6.68 Hz, 1H), 7.33 (t, J = 6.68 Hz, 1H), 7.29 (d, J = 1.43 Hz, 1H), 2.92 (bs, 3H), 2.73 (m, 1H), 2.42 (bs, 4H), 1.72 (bs, 4H), LCMS for C_18_H_19_BrFN_5_: 404 [M + H]^+^.

#### 4.1.7 General Procedure for the Synthesis of IK1–1K5

The solution of **1I** (100 mg, 0.2473 mmol) and different substituted boronic acids (0.4946 mmol) in 1,4‐dioxane:water (4:1, 5 mL) was kept for stirring, and K_2_CO_3_ (102 mg, 0.7419 mmol) was added. The reaction mixture was purged with nitrogen to remove all air for 15 min, followed by adding Pd(dppf)Cl_2_. DCM complex (10 mg, 0.012 mmol) and purging with nitrogen again for 5 min. The resulting reaction mixture was heated at 100°C for 16 h. The progress of the reaction was monitored through TLC and LCMS. After completion of the reaction, water was added to the reaction mixture, and the product was extracted using ethyl acetate (100 mL X 3). The combined organic layers were dried over anhydrous Na_2_SO_4_ and concentrated under a vacuum using a rotary evaporator to give a crude product. The crude was purified by flash or column chromatography (5% MeOH in DCM) as per the requirement to yield the final target compounds **1K1‐1K5** in good yield. The final products were characterized using LCMS and NMR. The HRMS, NMR, and UPLC purity graphs are available in the supplementary file S1.

##### 4.1.7.1 *N*‐(3‐Fluoro‐4‐(1‐Methylpiperidin‐4‐yl)phenyl)‐6‐(3,4,5‐trifluorophenyl)pyrrolo[2,1‐*f*][1,2,4]Triazin‐4‐Amine (1K1)

Off‐white solid, Yield: 55%, UPLC Purity: 99.78%, ^1^H NMR (400 MHz, DMSO‐d6): δ ppm 8.51 (s, 1H), 8.08 (s, 1H), 7.85 (d, *J* = 8 Hz, 1H), 7.80–7.76 (m, 1H), 7.52 (s, 1 H), 7.19–7.26 (m, 2H), 6.99 (s, 1H), 6.86 (s, 1H), 2.50 (m, 3H), 2.38 (m, 2H), 1.89 (m, 4H), 1.25–1.41 (m, 3H), ^13^C NMR (100 MHz, DMSO‐d6): δ = 161.88, 152.09, 147.76, 136.76, 129.61, 128.10, 128.04, 124.57, 117.34, 116.75, 115.83, 110.63, 110.10, 109.99, 109.94, 109.64, 109.36, 98.65, 96.66, 56.31, 46.95, 34.40, 32.15, 29.83, LCMS for C_24_H_21_F_4_N_5_: Calculated: 455.2, observed for [M + H]^+^: 456.3. HRMS calculated for C_24_H_21_F_4_N_5_: 455.1733, observed 456.1828 [M + H]^+^.

##### 4.1.7.2 6‐(3‐Chloro‐4‐Ethoxy‐5‐Methoxyphenyl)‐*N*‐(3‐Fluoro‐4‐(1‐Methylpiperidin‐4‐yl)phenyl)pyrrolo[2,1‐*f*][1,2,4]Triazin‐4‐Amine (1K2)

Off‐white solid, Yield: 57%, UPLC Purity: 99.76%, ^1^H NMR (400 MHz, DMSO‐*d*_6_): δ ppm 9.96 (s, 1H), 8.42 (s, 1H), 8.15 (s, 1H), 8.08 (s, 1H), 7.92 (d, *J* = 12.40 Hz, 1H), 7.57 (d, *J* = 7.63 Hz, 1H), 7.51 (s, 1H), 7.32–7.39 (m, 3H), 4.03 (q, *J* = 6.68 Hz, 2H), 3.93 (s, 3H), 2.92 (d, *J* = 10.97 Hz, 3H), 2.24 (bs, 2H), 2.08 (m, 1H), 1.72 (bs, 2H), 1.31 (t, *J* = 6.68 Hz, 3H), 1.23(bs, 4H) ^13^C NMR (100 MHz, DMSO‐d6): δ = 161.75, 159.32, 154.47, 152.01, 147.46, 143.98, 137.50, 137.38, 130.61, 129.30, 128.22, 128.07, 127.85, 127.79, 126.10, 119.63, 117.22, 117.19, 116.52, 115.66, 109.47, 109.19, 108.90, 59.52, 56.38, 56.03, 45.82, 33.83, 31.42, 15.70, LCMS for C_27_H_29_ClFN_5_O_2_: Calculated: 509.2, observed for [M + H]^+^: 510.3. HRMS calculated for C_27_H_29_ClFN_5_O_2_: 509.1994, observed 510.2086 [M + H]^+^; 512.2061 [M + 2]^+^.

##### 4.1.7.3 6‐(4‐(Tert‐butyl)phenyl)‐*N*‐(3‐Fluoro‐4‐(1‐Methylpiperidin‐4‐yl)phenyl)pyrrolo[2,1‐*f*][1,2,4]Triazin‐4‐Amine (1K3)

Off‐white solid, Yield: 51%, UPLC Purity: 99.88%, ^1^H NMR (400 MHz, DMSO‐*d*_6_): δ ppm 9.98 (s, 1H), 8.28 (s, 1H), 8.07 (s, 1H), 7.93 (d, *J* = 11.44 Hz, 1H), 7.67 (d, *J* = 8.11 Hz, 2H), 7.59 (d, *J* = 8.11 Hz, 1H), 7.51 (bs, 1H), 7.45 (d, *J* = 8.11 Hz, 2H), 7.33 (t, *J* = 8.11 Hz, 1H), 2.99 (bs, 2H), 2.76 (bs, 2H), 2.33 (bs, 2H), 1.76 (bs, 4H), 1.31 (s, 9H), 1.23 (bs, 2H), ^13^C NMR (100 MHz, DMSO‐d6): δ = 161.65, 151.98, 150.42, 147.19, 137.90, 137.78, 131.20, 127.68, 127.62, 127.25, 126.04, 125.81, 117.25, 116.46, 115.55, 109.41, 109.13, 97.21, 55.93, 45.75, 34.71, 33.45, 32.06, 31.45, 30.96, 29.83, 29.64, 29.49, 22.82, 14.25, LCMS for C_28_H_32_FN_5_: Calculated: 457.3, observed for [M + H]^+^: 458.4. HRMS calculated for C_28_H_32_FN_5_: 457.2642, observed 458.2731[M + H]^+^.

##### 4.1.7.4 6‐(3‐Chloro‐4‐Fluoro‐5‐Methoxyphenyl)‐*N*‐(3‐Fluoro‐4‐(1‐Methylpiperidin‐4‐yl)phenyl)pyrrolo[2,1‐*f*][1,2,4]Triazin‐4‐Amine (1K4)

Off‐white solid, Yield: 54%, UPLC Purity: 99.34%, ^1^H NMR (400 MHz, DMSO‐*d*_6_): δ ppm 8.46 (d, *J* = 1.43 Hz, 1H), 8.18 (s, 1H), 8.09 (s, 1H), 7.93 (dd, *J* = 12.87, 1.91 Hz, 1H), 7.53–7.59 (m, 2H), 7.47 (t, *J* = 6.20 Hz, 2H), 7.34 (t, *J* = 8.58 Hz, 1H), 3.98 (s, 3H), 2.92 (d, *J* = 11.44 Hz, 2H), 2.67–2.74 (m, 1H), 2.24 (s, 3H), 2.03–2.09 (m, 2H), 1.71–1.76 (m, 4H), ^13^C NMR (100 MHz, DMSO‐d6): δ = 161.72, 151.89, 149.06, 148.95, 147.44, 146.76, 136.88, 136.77, 130.57, 127.91, 127.85, 125.52, 122.31, 122.16, 119.43, 117.11, 116.55, 115.53, 109.72, 109.42, 109.14, 96.61, 56.73, 56.07, 46.13, 34.11, 31.77, LCMS for C_25_H_24_ClF_2_N_5_O: Calculated: 483.2, observed for [M + H]^+^: 484.2. HRMS calculated for C_25_H_24_ClF_2_N_5_O: 483.1637, observed 484.1729[M + H]^+^; 486.1695 [M + 2]^+^.

##### 4.1.7.5 6‐(4‐Ethoxy‐3‐Methoxyphenyl)‐*N*‐(3‐Fluoro‐4‐(1‐Methylpiperidin‐4‐yl)phenyl)pyrrolo[2,1‐*f*][1,2,4]Triazin‐4‐Amine (1K5)

Off‐white solid, Yield: 56%, UPLC Purity: 99.85%, ^1^H NMR (400 MHz, DMSO‐*d*_6_): δ ppm 8.29 (d, *J* = 1.43 Hz, 1H), 8.16 (s, 1H), 8.06 (s, 1H), 7.93 (dd, *J* = 13.35, 1.91 Hz, 1H), 7.58 (d, *J* = 8.58 Hz, 1H), 7.46 (d, *J* = 1.43 Hz, 1H), 7.30–7.36 (m, 2H), 7.24 (d, *J* = 8.11 Hz, 1H), 7.01 (d, *J* = 8.58 Hz, 1H), 4.04 (q, *J* = 7.15 Hz, 2H), 3.86 (s, 3H), 2.91 (d, *J* = 10.97 Hz, 2H), 2.73 (bs, 1H), 2.24 (s, 3H), 2.04 (m, 2H), 1.72 (m, 4H), 1.34 (t, *J* = 8.58 Hz, 3H), ^13^C NMR (100 MHz, DMSO‐d6): δ = 169.33, 161.45, 159.03. 151.97, 149.68, 147.80, 147.19, 138.75, 138.63, 127.33, 127.27, 127.12, 127.02, 125.99, 125.84, 118.39, 117.01, 115.85, 115.76, 113.23, 109.65, 109.18, 108.89, 97.66, 64.52, 56.14, 55.20, 44.41, 32.84, 30.08, 14.91, LCMS for C_27_H_30_FN_5_O_2_: Calculated: 475.2, observed for [M + H]^+^: 476.3. HRMS calculated for C_27_H_30_FN_5_O_2_: 475.2384, observed 476.2474 [M + H]^+^.

### 4.2 Biological Evaluation

#### 4.2.1 Cell Viability Studies

The cell viability assay of new compounds was performed against A549, MCF‐7, and MDA‐MB‐231 carcinoma cell lines. The cells (2500 cells/well) were seeded in a 96‐well plate using DMEM media (Gibco by Life Technologies) consisting of 10% FBS and subjected to incubation at 37°C for 24 h in 5% CO_2_ in a Nuaire incubator (humidified) for adherence to surface and growth. After 24 h, cells were subjected to treatment against target compounds (1K1‐1K5) at different concentrations (final concentrations ranging from 0.01–30 μM) and left for incubation for 72 h at the same conditions as mentioned above. The same procedure was repeated after 72 h after replacing the remaining test compound with fresh media and incubating treated cells for a further 48 h. After incubation, the remaining compounds with media were removed followed by washing with fresh media and the addition of 100 μL of resazurin (1 mM, Sigma). The 96‐well plate was incubated for 4 h at room temperature. A multimode microplate reader was used to record fluorescence at 535 nm (excitation) and 590 nm (emission) wavelengths. Background fluorescence was subtracted for calculating final results and avoiding any interference and normalized against vehicle control (DMSO treated cells) to calculate final cell viability. Cell viability (percentage) was used to calculate the IC_50_ values of the target compounds using fitting the curve to the “four‐parameter variable slope logistic model” using Prism Graph Pad (Kathuria et al. 2023; Rani et al. 2023).

#### 4.2.2 DMPK Studies

The DMPK evaluation involves multiple reagents and instruments. The chemical and reagents include synthesized target molecules (at a concentration of 1–3 mg), DMSO (HPLC grade), PBS (pH = 7.4), NADPH, and liver microsomes (human and mouse) procured from Thermo. The instruments include Nexera X2 UPLC with an AB Sciex QTrap 4500 MS/MS detector (Shimadzu), single and multichannel micropipette (Eppendorf USA and Thermo), a 96‐well plate (1 mL capacity with deep wells) vortex mixer (Tarsons), centrifuge tubes (Tarsons) water bath (MRC), and a centrifuge (Thermo). The fresh stock solutions (10 mM) were prepared and incubations were carried out in duplication. Each well‐comprised potassium phosphate buffer (100 mM, pH 7.4) with 0.5 mg/mL microsome, NADPH (1 mM), and target molecule (1 μM). After 30 min incubation (37°C), the aliquots were taken at 0 and 30 min and quenched with chilled acetonitrile comprising Terfenadine as internal standard. The samples were centrifuged at 4000 rpm at 4°C, and the supernatant was subjected to LC–MS/MS for analysis. The experiment was used to determine the half‐life of the compound with the help of peak area ratio relative to the internal standard.

#### 4.2.3 MERTK Inhibitory Activity

ELISA kit (z‐Lyte kinase assay kit‐tyr4 peptide) was utilized to determine MERTK inhibitory activity as per the protocol supplied by the supplier along with the kit. Briefly, the mixture of MERTK (cMER) / Tyr 02 (2X) was made using HEPES buffer (50 mM, pH 7.5) comprising polyoxyethylene lauryl ether solution (0.01%), MgCl_2_ (10 mM), MnCl_2_ (4 mM), EGTA (1 mM), and DTT (2 mM). The kinase solution of 10 μL was used as the final reaction solution. In the next step, the target molecules in varying concentrations were incubated with kinase solution for 1 h. Development reagent A (5 μL) of 1:128 dilution was added after the incubation period. The fluorescence was measured at Ex/Em 400, 445, and 520 nm using a microplate reader for the determination of final percentage inhibition. The percent phosphorylation was determined via the use of the emission ratio calculated from the division of Coumarin Emission (at 445 nm) to fluorescein emission (at 520 nm).

$$\begin{array}{c} \%\text{phosphorylation}=1-\frac{\left(\text{Emission ratio}\;\times \;F100\%\right)-C100\%}{\left(C0\%-C100\%\right)+\left[\text{Emission ratio}\;\left(F100\%-F0\%\right)\right]} \end{array}$$

*F*100% = Average Fluorescein emission signal of the 100% Phos. Control.

*C*100% = Average coumarin emission signal of the 0% Phos. Control.

*C*0% = Average coumarin emission signal of the 100% Phos. Control.

*F*0% = Average Fluorescein emission signal of the 0% Phos. Control (Kalra et al. 2020).

### 4.3 In Silico Studies

The target molecules were subjected to molecular docking studies against MERTK (PDB ID: 7AAY) using Schrodinger Maestro software as per previous studies performed by us (Kumar, Kumar, and Bhatia 2023; Kumar et al. 2018). The MERTK (PDB ID: 7AAY) X‐ray structure was downloaded from www.rcsb.org (Pflug et al. 2020). “Protein preparation wizard” was employed to prepare the molecule in which energy minimization was done using forcefield OPLS2005. Using a grid generation module, the docking grid was generated around the co‐crystallized ligand. Initially drawn 2D structures of target molecules prepared using “Ligprep” module for final docking at prepared protein. A Glidedock module was employed for docking ligands and protein, which was validated via re‐docking of co‐crystallized ligands at the binding site of the target protein and observed RMSD values. The top dock score pose was employed for visualization of the XP interaction visualizer for evaluating ligand–protein interactions. The in silico drug‐likeness of synthesized ligands was predicted using Swiss ADME, a free online tool, while the toxicity profile was predicted using ProTox (Rawal et al. 2023). The MD analysis was performed using the Desmond module of Schrodinger software (Release 2019). The best‐identified ligand within the catalytic site of protein was simulated for 100 ns to confirm the stability.

## Conflicts of Interest

The authors declare no conflicts of interest.

## Supporting information


**Data S1.** The analytical data of the synthesized compounds, including HRMS, NMR, and UPLC purity graphs, are available in the supplementary information.

## Data Availability

The data that supports the findings of this study are available in the supplementary material of this article.
