# Supplementary material for: Design and Development of Novel Hybrids Based on Pyrrolo[2,1‐f][1,2,4]Triazine and 1‐(Methylpiperidin‐4‐yl) Aniline–Based Analogs: Exploring the Utility as Anticancer Agents via MERTK Inhibition
Source: Chem Biol Drug Des. 2024 Dec 5;104(6):e70023. doi: 10.1111/cbdd.70023 (PMC11618975; doi:10.1111/cbdd.70023)
Supplement: Supplementary file 1 — Data S1. The analytical data of the synthesized compounds, including HRMS, NMR, and UPLC purity graphs, are available in the supplementary information. [file CBDD-104-e70023-s001.pdf]

Design and development of novel hybrids based on pyrrolo[2,1-*f*][1,2,4]triazine and 1-(methylpiperidin-4-yl) aniline-based analogues: Exploring the utility as anticancer agents via MERTK inhibition

Balaji Dashrath Sathe,[1,2] Shivani Jaiswal,[3] Devendra Kumar,[4] Thakur Gurjeet Singh,[5] Nidhi Nainwal,[6] Pramod Rawat,[7,8] Savita Yadav,[9] Bhupinder Kumar,[10] Ashish Ranjan Dwivedi,[11*] S.V. Rathod,[1*]

[1]Department of Chemistry, Bharatiya Vidya Bhavan College, Chowpatty, Mumbai University, Mumbai, India. 400 007

[2]Integral Biosciences Pvt Ltd., Drug Discovery Biotech, Delhi NCR-Noida, India 201306

[3]Institute of Pharmaceutical Research, GLA University Mathura-281406, India

[4]School of Pharmacy, Narsee Monjee Institute of Management Studies (NMIMS) Dist. Dhule, Maharashtra, India.

[5]Centre for Research Impact & Outcome, Chitkara College of Pharmacy, Chitkara University, Rajpura, 140401, Punjab, India

[6]Uttaranchal Institute of Pharmaceutical Sciences, Uttaranchal University, Premanagar, Dehradun 248007, Uttarakhand, India

[7]Graphic Era (Deemed to be University) Clement Town Dehradun- 248002, India

[8]Graphic Era Hill University Clement Town Dehradun- 248002, India

[9]IES Institute of Pharmacy, IES University, Bhopal, Madhya Pradesh 462044, India

[10]Department of Pharmaceutical Sciences, Chauras Campus, HNB Garhwal University (A Central University), Srinagar, Uttarakhand 246174, India.

[11]GITAM School of Pharmacy, GITAM (Deemed to be) University, Hyderabad, 502329, India

***Corresponding Author:**

Dr Ashish Ranjan Dwivedi

Assistant Professor, GITAM School of Pharmacy, GITAM (Deemed to be) University, Hyderabad-502329. India; Email: ashishrdwdy@gmail.com

Prof. (Dr.) S.V. Rathod

Department of Chemistry, Bharatiya Vidya Bhavan College, Chowpatty, Mumbai University, Mumbai, India. 400 007

Email: shrees.rathod@gmail.com

1K1

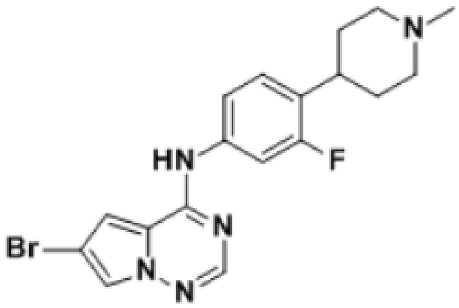

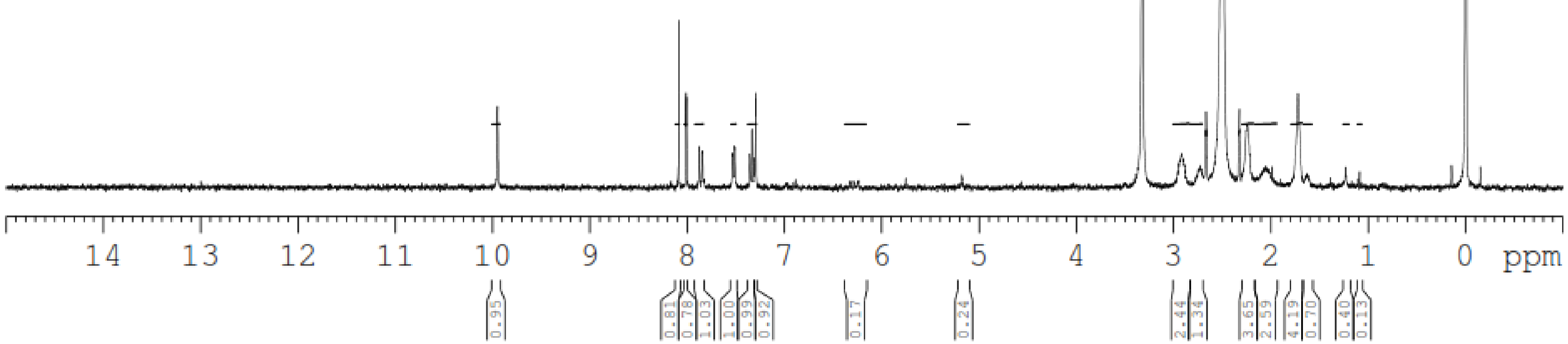

Chemical Formula: $C_{18}H_{19}BrFN_5$
Exact Mass: 403.08
Molecular Weight: 404.28

```
Current Data Parameters
NAME      BS-IBS-023-007-Y
EXPNO                   33
PROCNO                   1

F2 - Acquisition Parameters
Date_             20230105
Time                 14.26 h
INSTRUM          AvanceNeo
PROBHD     Z163739_0475 (
PULPROG               zg30
TD                   32768
SOLVENT               DMSO
NS                       8
DS                       0
SWH               7812.500 Hz
FIDRES            0.476837 Hz
AQ               2.0971520 sec
RG                     101
DW                  64.000 usec
DE                    6.64 usec
TE                   295.7 K
D1              1.00000000 sec
TD0                      1
SFO1           399.7884687 MHz
NUC1                    1H
P0                    2.67 usec
P1                    8.00 usec
PLW1           22.80999947 W

F2 - Processing parameters
SI                   16384
SF             399.7860029 MHz
WDW                     EM
SSB                      0
LB                    0.30 Hz
GB                       0
PC                    1.00
```

1K1

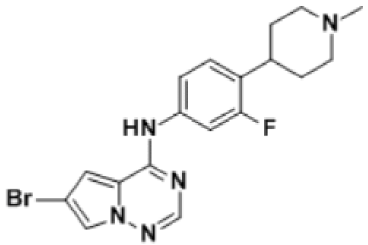

Chemical Formula: $C_{18}H_{19}BrFN_5$
Exact Mass: 403.08
Molecular Weight: 404.28

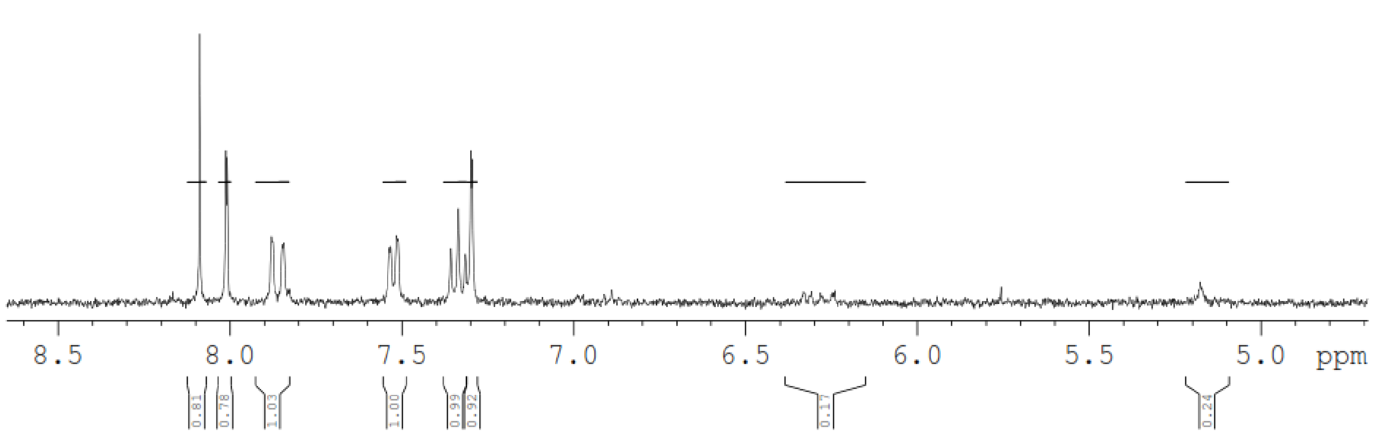


```
Current Data Parameters
NAME       BS-IBS-023-007-Y
EXPNO                    33
PROCNO                    1

F2 - Acquisition Parameters
Date_              20230105
Time                  14.26 h
INSTRUM           AvanceNeo
PROBHD    Z163739_0475 (
PULPROG                zg30
TD                    32768
SOLVENT                DMSO
NS                        8
DS                        0
SWH                7812.500 Hz
FIDRES             0.476837 Hz
AQ                2.0971520 sec
RG                      101
DW                   64.000 usec
DE                     6.64 usec
TE                    295.7 K
D1               1.00000000 sec
TD0                       1
SFO1            399.7884687 MHz
NUC1                     1H
P0                     2.67 usec
P1                     8.00 usec
PLW1            22.80999947 W

F2 - Processing parameters
SI                    16384
SF              399.7860029 MHz
WDW                      EM
SSB                       0
LB                     0.30 Hz
GB                        0
PC                     1.00
```

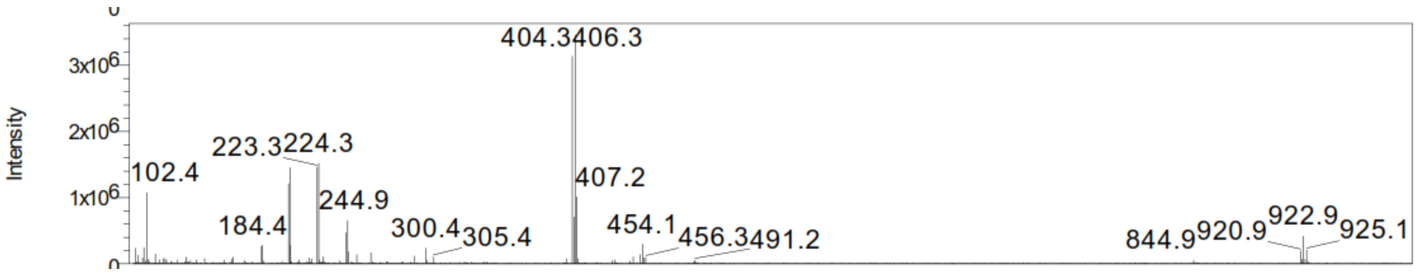

BS-IBS-023-008-F

1K1

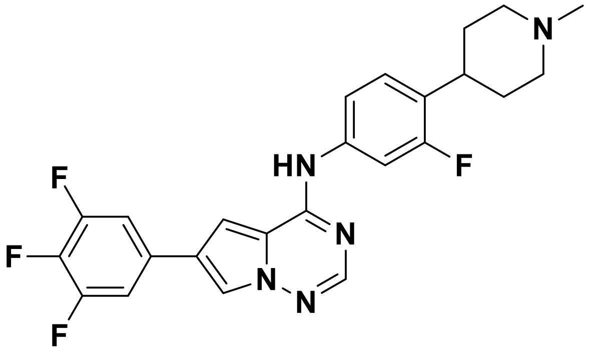

1K1

Chemical Formula: $C_{24}H_{21}F_4N_5$

Exact Mass: 455.1733

Molecular Weight: 455.4606

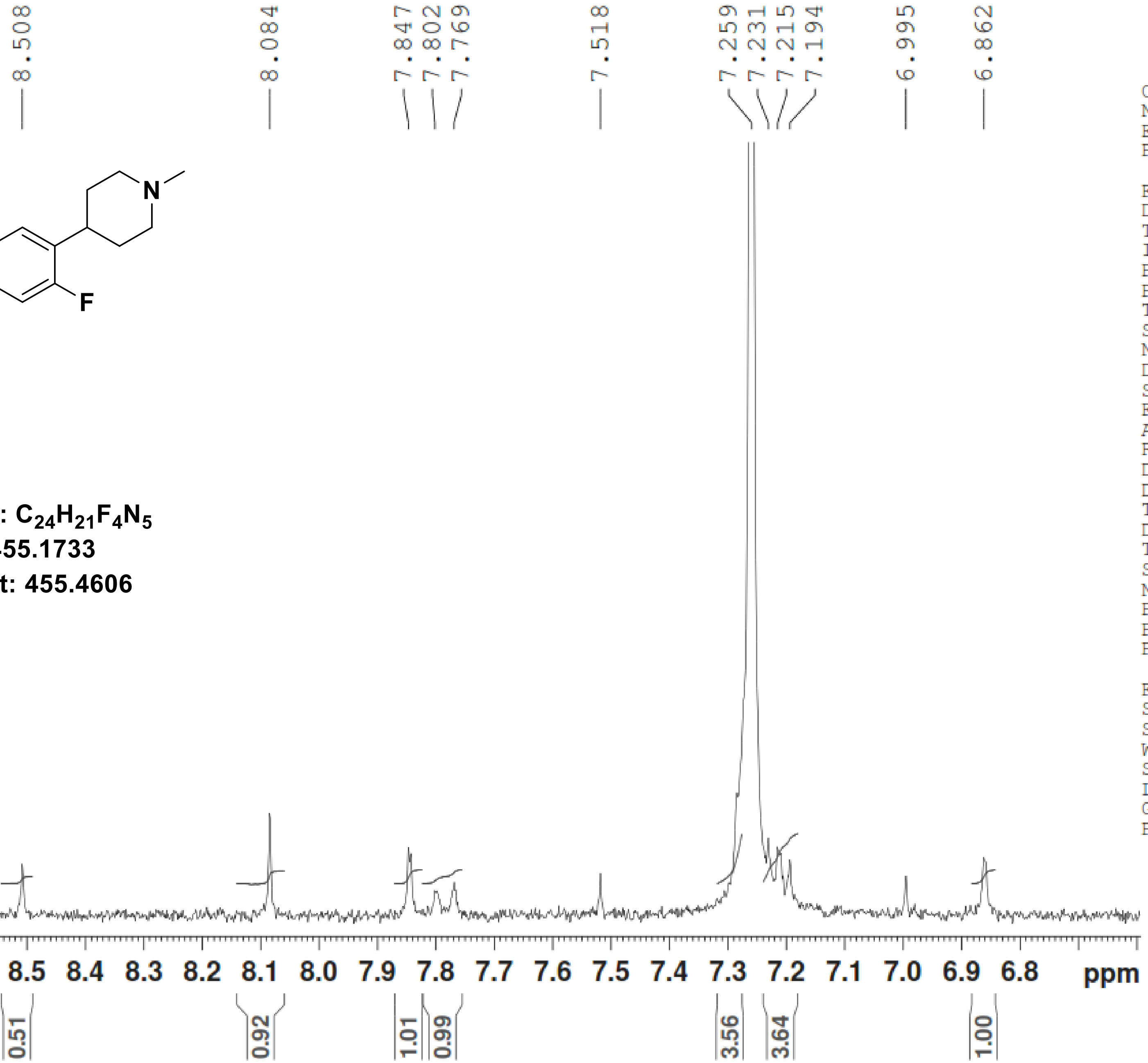


```
Current Data Parameters
NAME     BS-IBS-023-008-F
EXPNO                  10
PROCNO                  1

F2 - Acquisition Parameters
Date_            20230118
Time                15.27 h
INSTRUM         AvanceNeo
PROBHD   Z163739_0475 (
PULPROG              zg30
TD                  32768
SOLVENT             CDCl3
NS                      8
DS                      0
SWH              7812.500 Hz
FIDRES           0.476837 Hz
AQ              2.0971520 sec
RG                    101
DW                 64.000 usec
DE                   6.64 usec
TE                  295.2 K
D1             1.00000000 sec
TD0                     1
SFO1          399.7884687 MHz
NUC1                   1H
P0                   2.67 usec
P1                   8.00 usec
PLW1          22.80999947 W

F2 - Processing parameters
SI                  16384
SF            399.7860102 MHz
WDW                    EM
SSB                     0
LB                   0.30 Hz
GB                      0
PC                   1.00
```

BS-IBS-023-008-C13

1K1

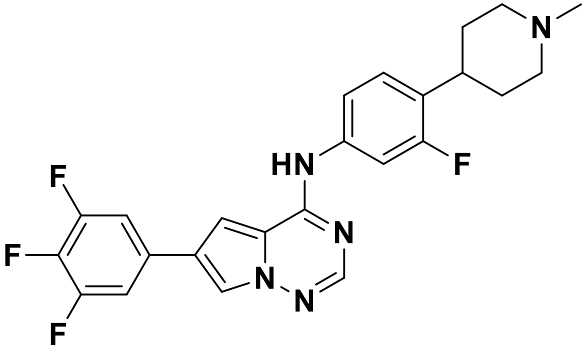

1K1
Chemical Formula: $C_{24}H_{21}F_4N_5$
Exact Mass: 455.1733
Molecular Weight: 455.4606

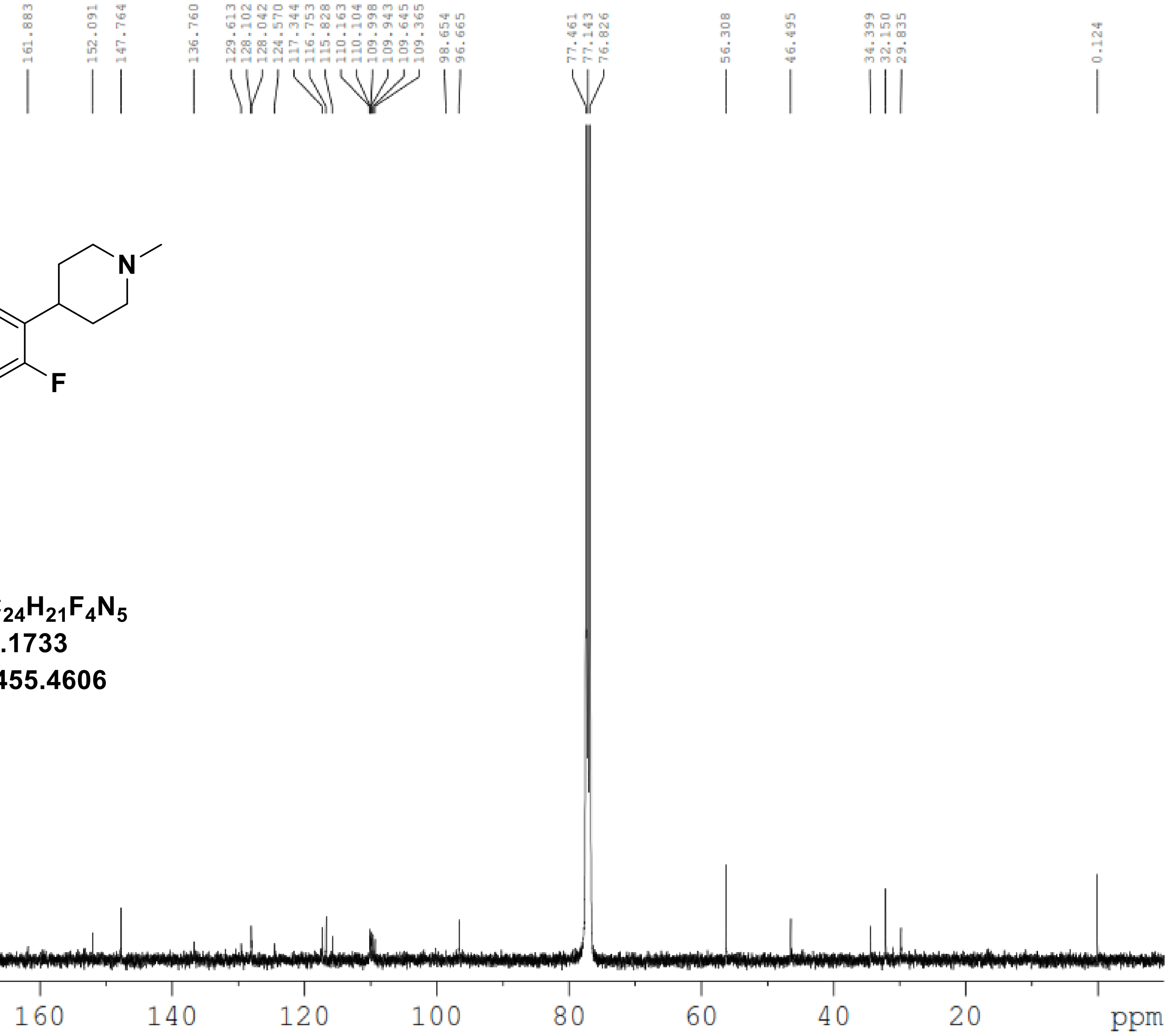

Current Data Parameters
NAME BS-IBS-023-008-C13
EXPNO 10
PROCNO 1

F2 - Acquisition Parameters
Date_ 20230120
Time 8.23 h
INSTRUM AvanceNeo
PROBHD Z163739_0475 (
PULPROG zgpg30
TD 65536
SOLVENT CDCl3
NS 15360
DS 4
SWH 23809.523 Hz
FIDRES 0.726609 Hz
AQ 1.3762560 sec
RG 30.1786
DW 21.000 usec
DE 6.50 usec
TE 296.3 K
D1 2.00000000 sec
D11 0.03000000 sec
TD0 1
SFO1 100.5363223 MHz
NUC1 13C
P0 2.67 usec
P1 8.00 usec
PLW1 100.66999817 W
SFO2 399.7875991 MHz
NUC2 1H
CPDPRG[2 waltz65
PCPD2 90.00 usec
PLW2 22.80999947 W
PLW12 0.17502500 W
PLW13 0.08772253 W

F2 - Processing parameters
SI 32768
SF 100.5262572 MHz
WDW EM
SSB 0
LB 1.00 Hz
GB 0
PC 1.40

1K1

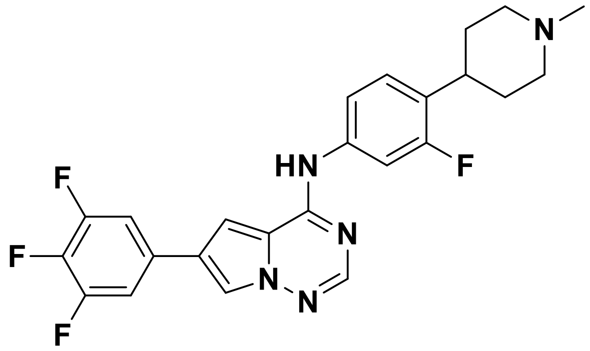

1K1
Chemical Formula: $C_{24}H_{21}F_4N_5$
Exact Mass: 455.1733
Molecular Weight: 455.4606

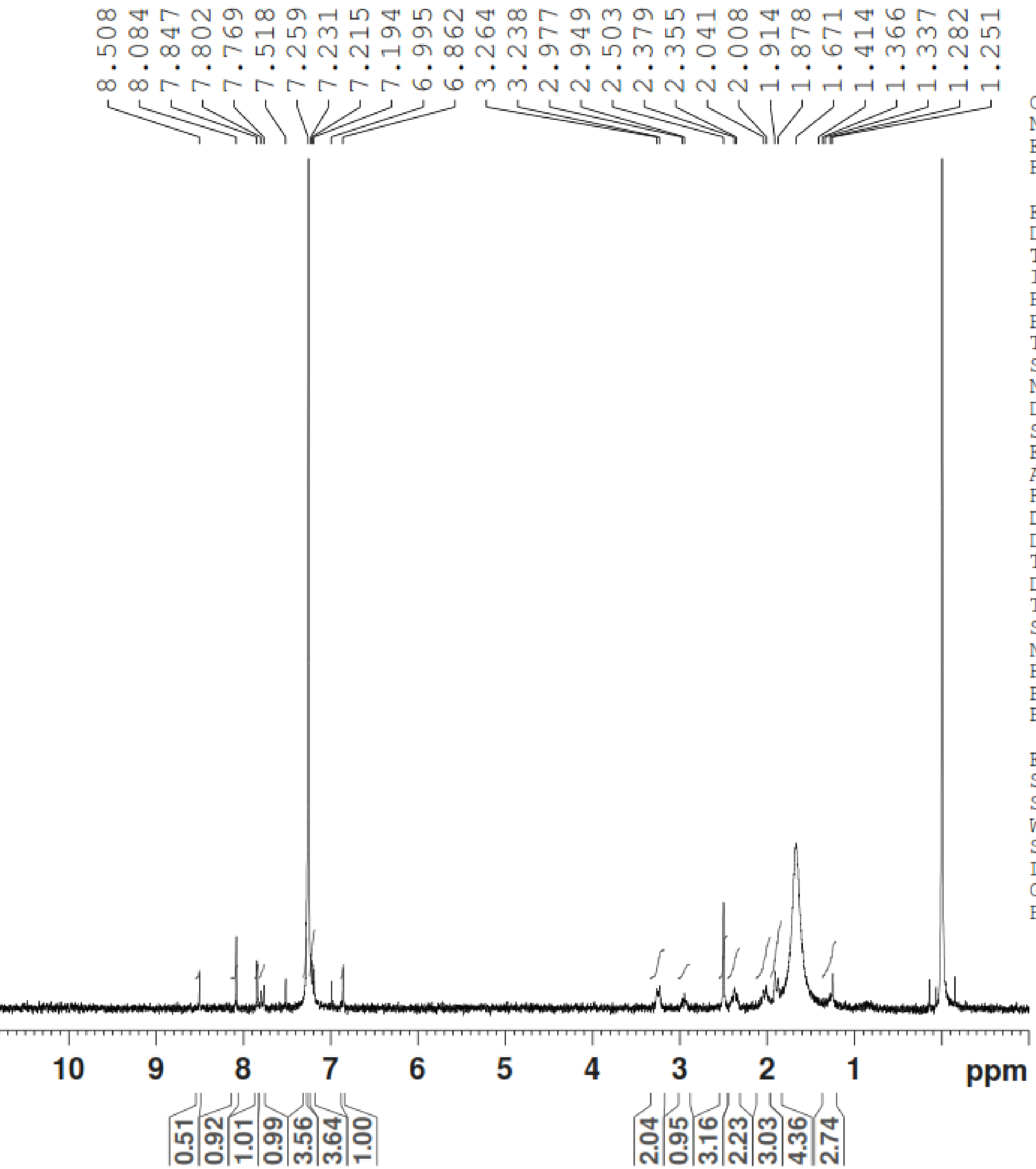

Current Data Parameters
NAME BS-IBS-023-008-F
EXPNO 10
PROCNO 1

F2 - Acquisition Parameters
Date_ 20230118
Time 15.27 h
INSTRUM AvanceNeo
PROBHD Z163739_0475 (
PULPROG zg30
TD 32768
SOLVENT CDCl3
NS 8
DS 0
SWH 7812.500 Hz
FIDRES 0.476837 Hz
AQ 2.0971520 sec
RG 101
DW 64.000 usec
DE 6.64 usec
TE 295.2 K
D1 1.00000000 sec
TD0 1
SFO1 399.7884687 MHz
NUC1 1H
P0 2.67 usec
P1 8.00 usec
PLW1 22.80999947 W

F2 - Processing parameters
SI 16384
SF 399.7860102 MHz
WDW EM
SSB 0
LB 0.30 Hz
GB 0
PC 1.00

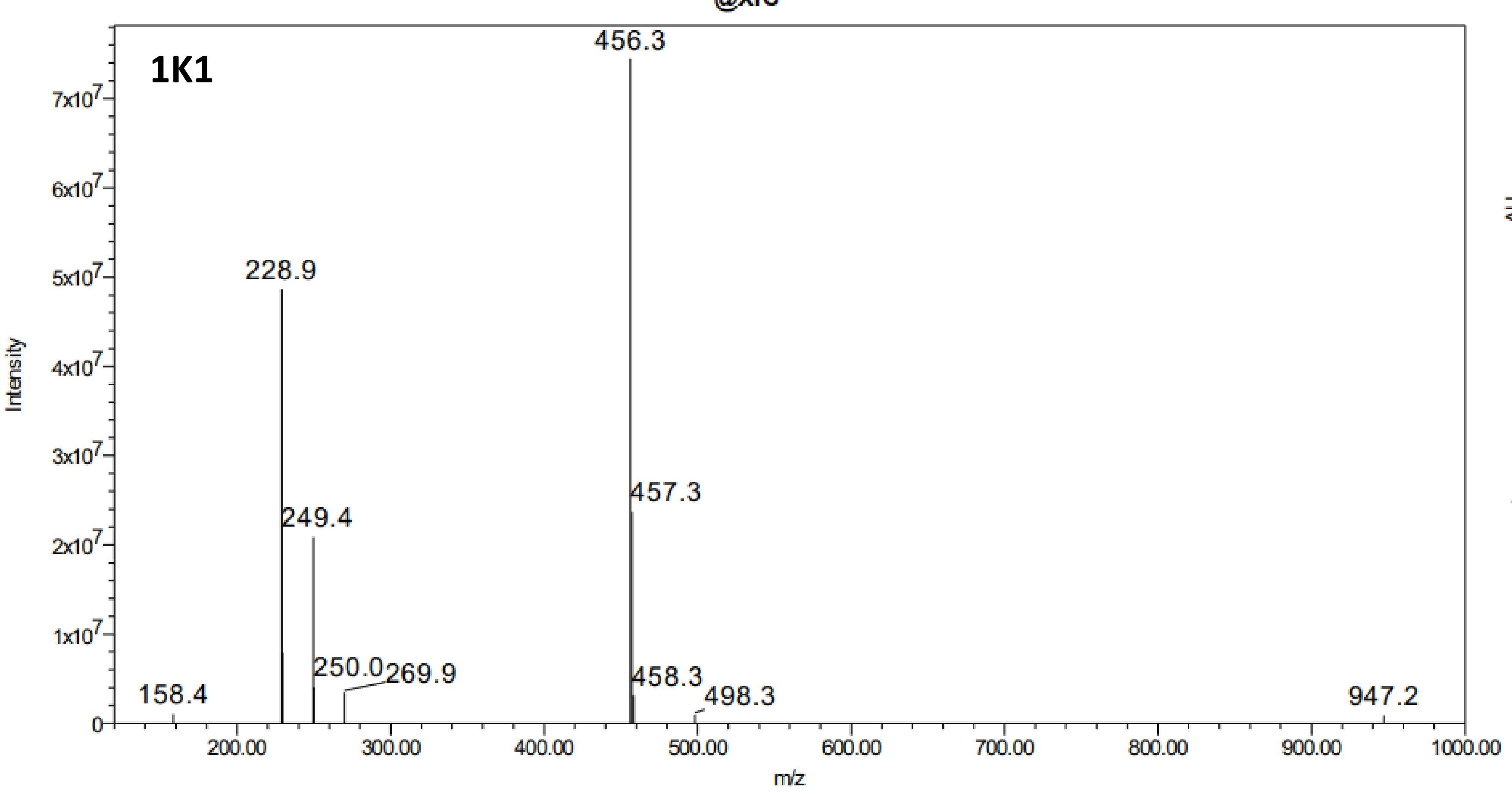

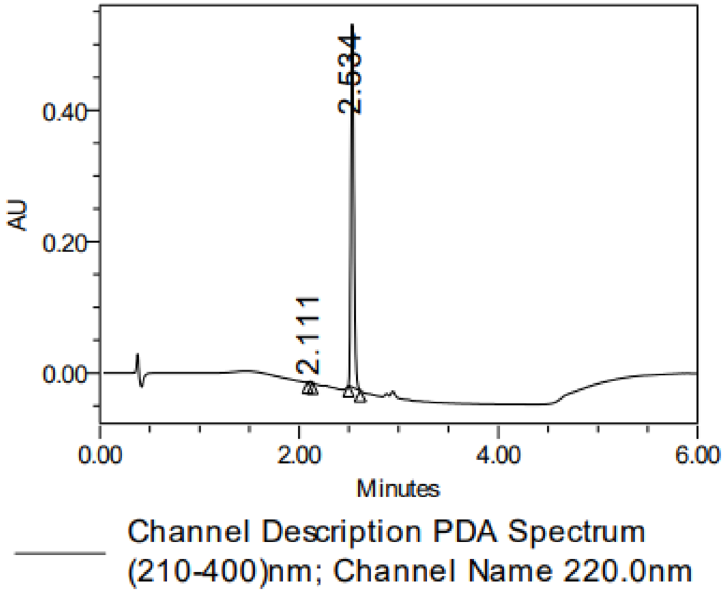

Processed Channel Descr.: PDA 220.0 nm (PDA Spectrum (210-400)nm)

	Processed Channel Descr.	Retention Time (min)	Area	% Area	Purity Angle	Purity Threshold
1	PDA 220.0 nm (PDA Spectrum (210-400)nm)	2.111	2643	0.22	2.33	3.29
2	PDA 220.0 nm (PDA Spectrum (210-400)nm)	2.534	1187425	99.78	0.20	0.25

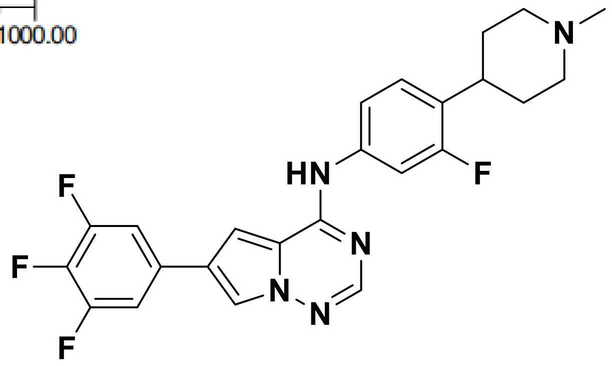

1K1

Chemical Formula: $C_{24}H_{21}F_4N_5$

Exact Mass: 455.1733

Molecular Weight: 455.4606

Sample Name : BDS-006
Test Name : HRMS-1

INDIAN INSTITUTE OF TECHNOLOGY
ROPAR

XEVO G2-XS QTOF

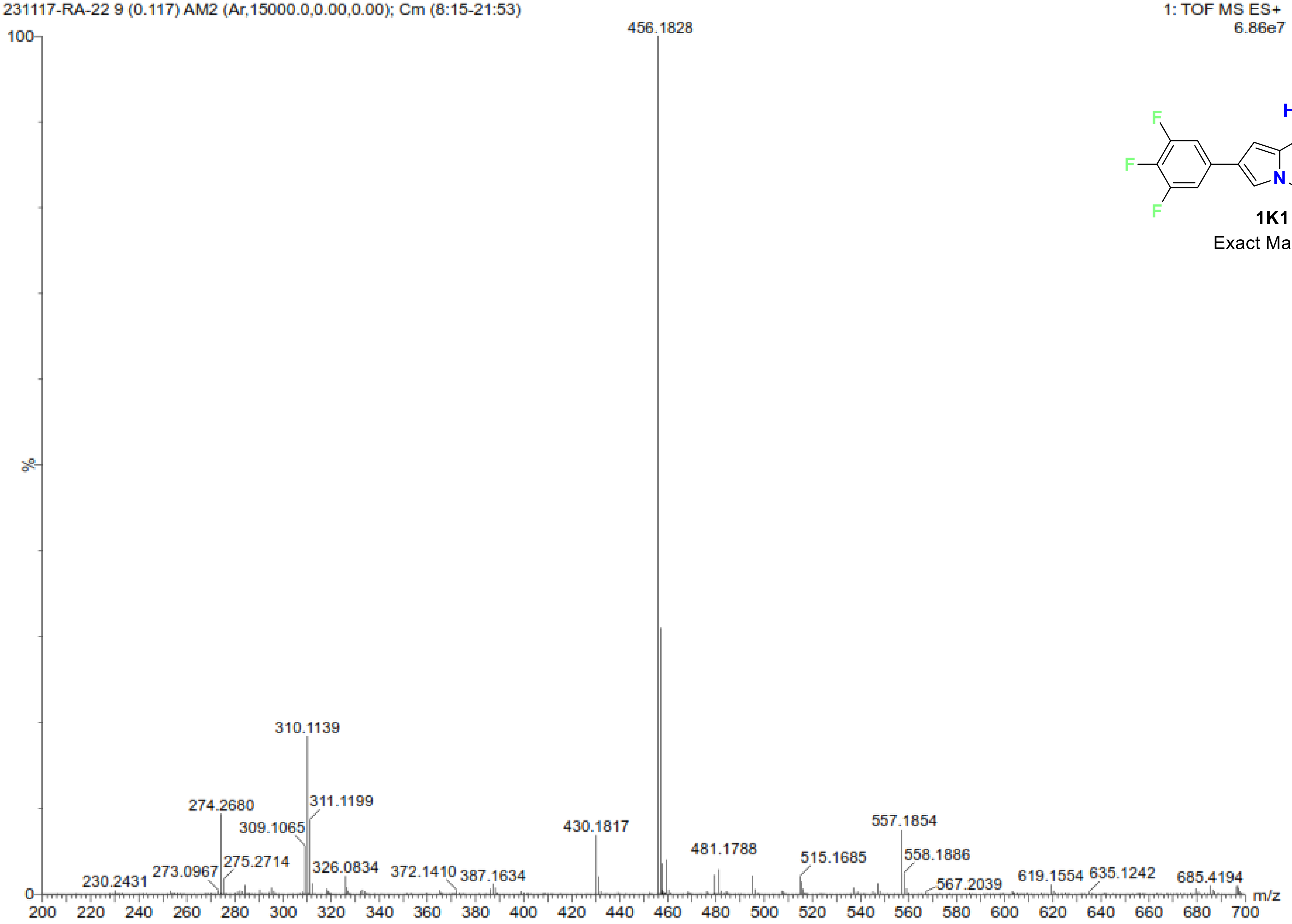

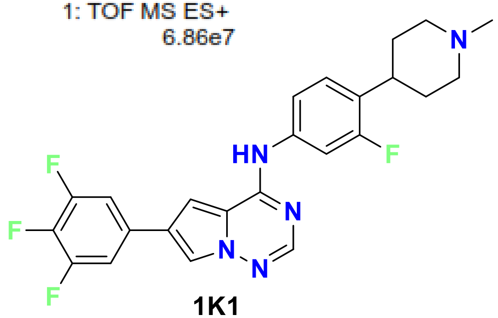

1K1

Exact Mass: 455.17

BS-IBS-023-009-F

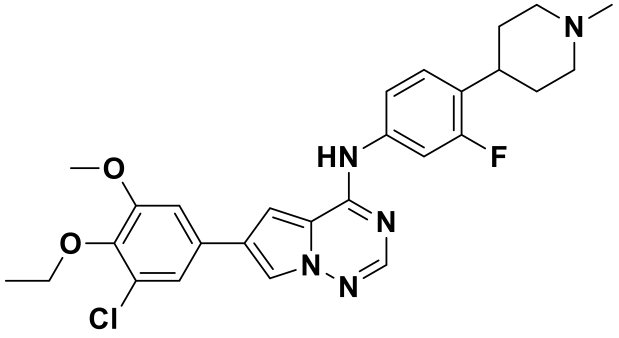

1K2

Chemical Formula: $C_{27}H_{29}ClFN_5O_2$

Exact Mass: 509.1994

Molecular Weight: 510.0104

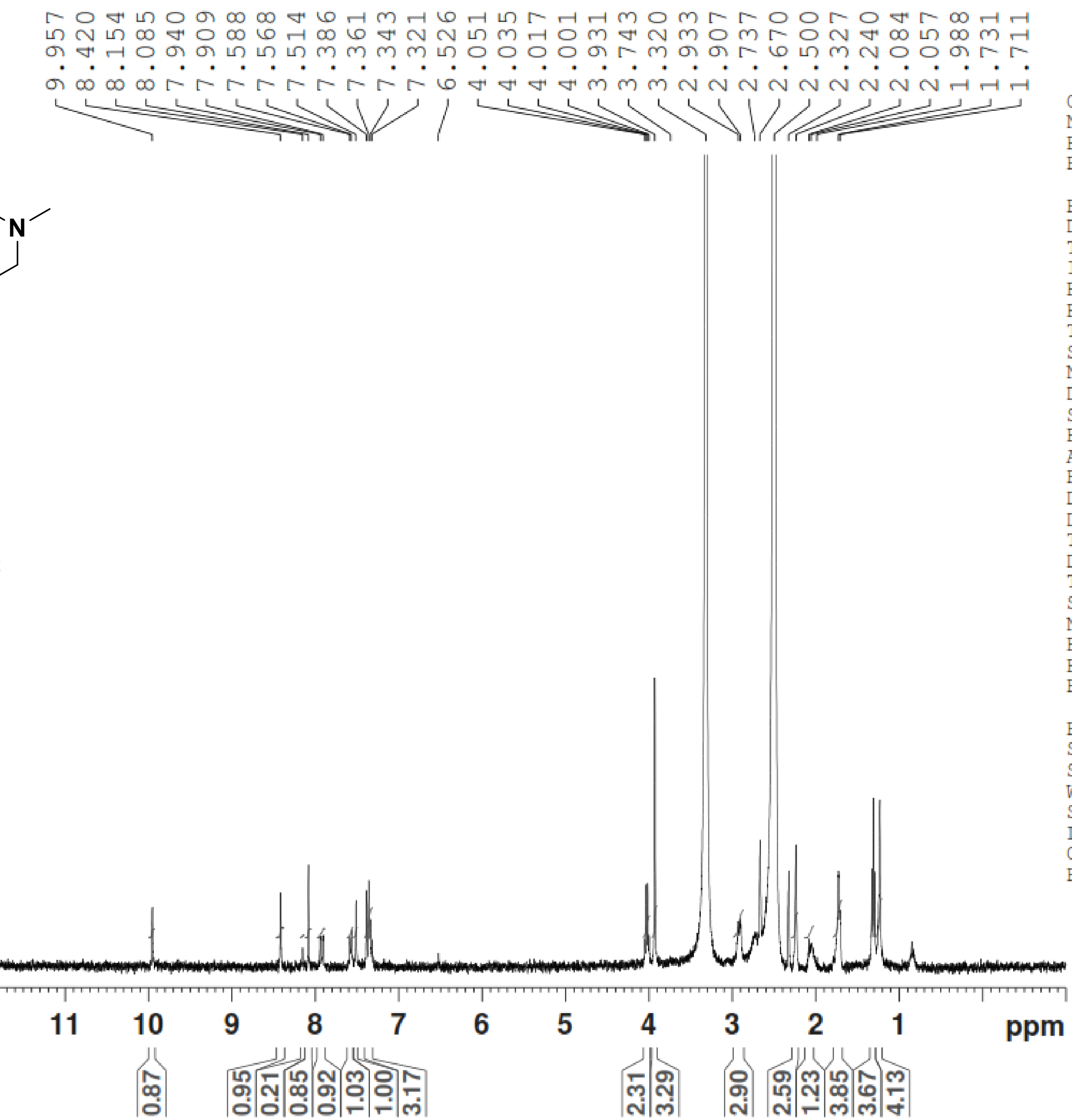


```
Current Data Parameters
NAME        BS-IBS-023-009-F
EXPNO                     18
PROCNO                     1

F2 - Acquisition Parameters
Date_               20230131
Time                 17.15 h
INSTRUM           AvanceNeo
PROBHD      Z163739_0475 (
PULPROG                 zg30
TD                     32768
SOLVENT                 DMSO
NS                         8
DS                         0
SWH                 7812.500 Hz
FIDRES              0.476837 Hz
AQ                 2.0971520 sec
RG                       101
DW                    64.000 usec
DE                      6.64 usec
TE                     297.4 K
D1                1.00000000 sec
TD0                        1
SFO1             399.7884687 MHz
NUC1                      1H
P0                      2.67 usec
P1                      8.00 usec
PLW1             22.80999947 W

F2 - Processing parameters
SI                     16384
SF               399.7860029 MHz
WDW                       EM
SSB                        0
LB                      0.30 Hz
GB                         0
PC                      1.00
```

BS-IBS-023-009-F

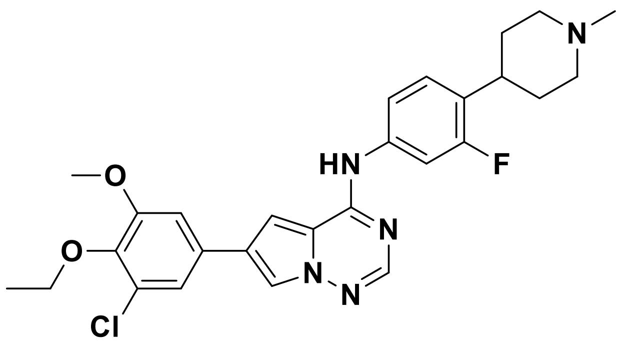

1K2

Chemical Formula: $C_{27}H_{29}ClFN_5O_2$

Exact Mass: 509.1994

Molecular Weight: 510.0104

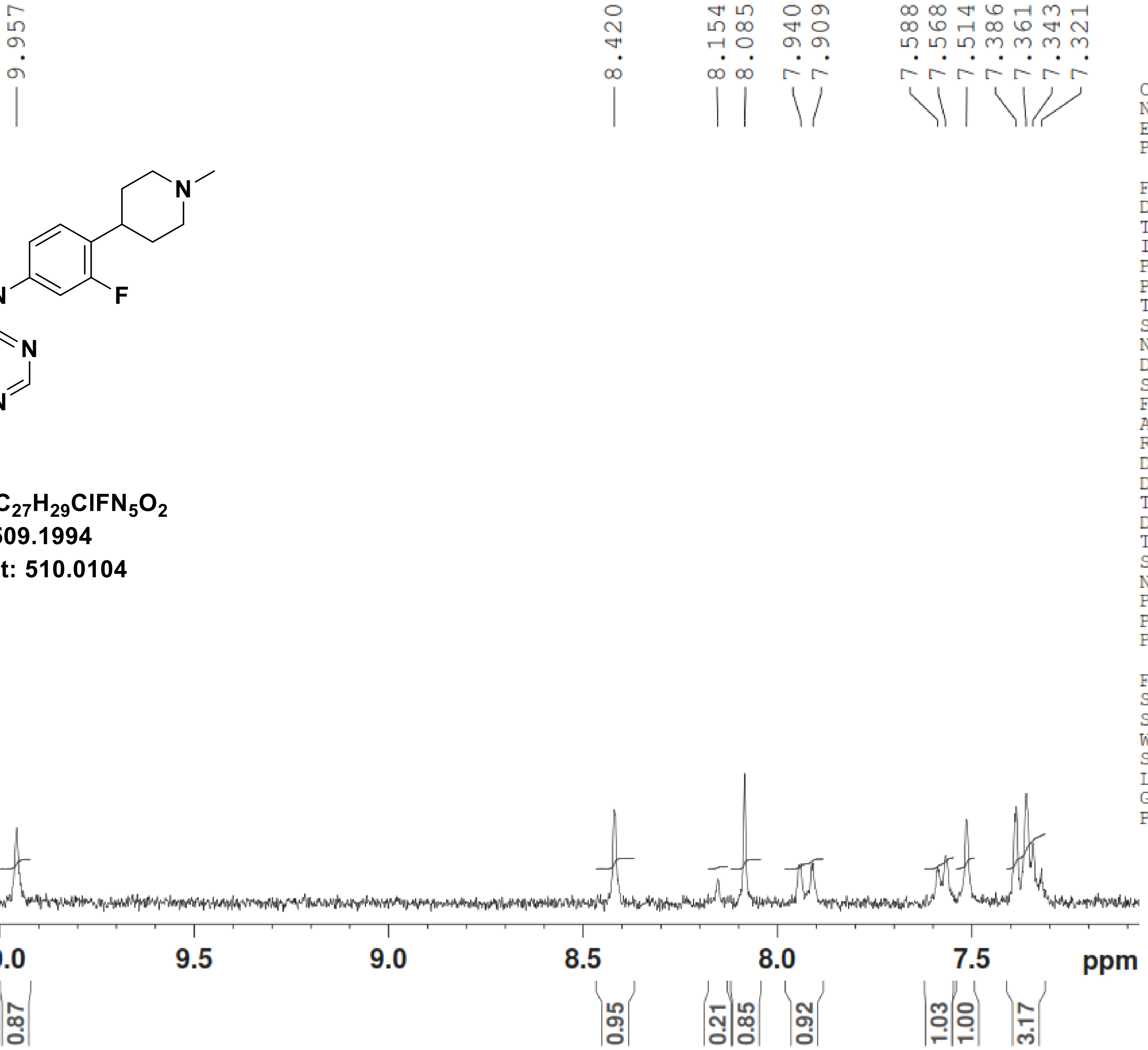

Current Data Parameters
NAME BS-IBS-023-009-F
EXPNO 18
PROCNO 1

F2 - Acquisition Parameters
Date_ 20230131
Time 17.15 h
INSTRUM AvanceNeo
PROBHD Z163739_0475 (
PULPROG zg30
TD 32768
SOLVENT DMSO
NS 8
DS 0
SWH 7812.500 Hz
FIDRES 0.476837 Hz
AQ 2.0971520 sec
RG 101
DW 64.000 usec
DE 6.64 usec
TE 297.4 K
D1 1.00000000 sec
TD0 1
SFO1 399.7884687 MHz
NUC1 1H
P0 2.67 usec
P1 8.00 usec
PLW1 22.80999947 W

F2 - Processing parameters
SI 16384
SF 399.7860029 MHz
WDW EM
SSB 0
LB 0.30 Hz
GB 0
PC 1.00

BS-IBS-023-009-F-13C

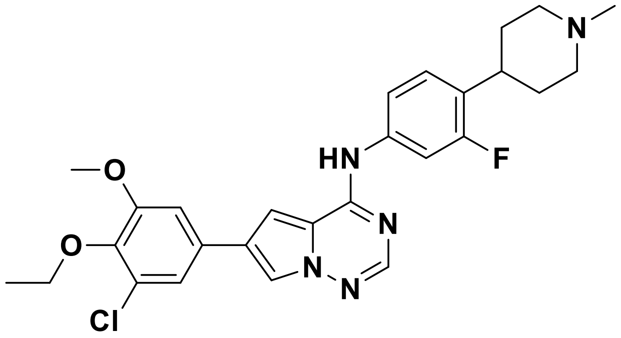

1K2

Chemical Formula: $C_{27}H_{29}ClFN_5O_2$

Exact Mass: 509.1994

Molecular Weight: 510.0104

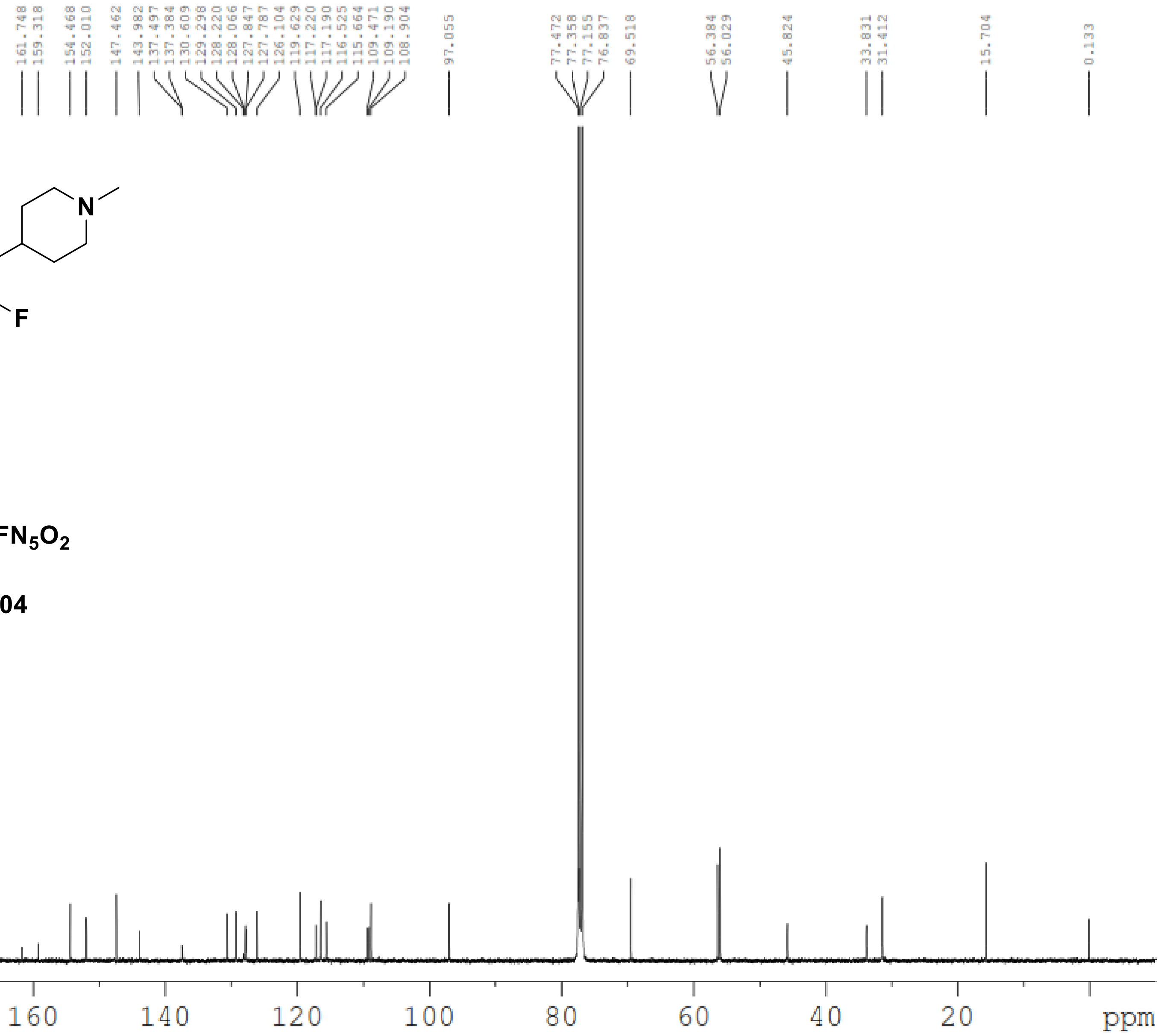

Current Data Parameters
NAME BS-IBS-023-009-F-13C
EXPNO 21
PROCNO 1

F2 - Acquisition Parameters
Date_ 20230204
Time 8.10 h
INSTRUM AvanceNeo
PROBHD Z163739_0475 (
PULPROG zgpg30
TD 65536
SOLVENT CDCl3
NS 15360
DS 4
SWH 23809.523 Hz
FIDRES 0.726609 Hz
AQ 1.3762560 sec
RG 47.2839
DW 21.000 usec
DE 6.50 usec
TE 297.2 K
D1 2.00000000 sec
D11 0.03000000 sec
TD0 1
SFO1 100.5363223 MHz
NUC1 13C
P0 2.67 usec
P1 8.00 usec
PLW1 100.66999817 W
SFO2 399.7875991 MHz
NUC2 1H
CPDPRG[2 waltz65
PCPD2 90.00 usec
PLW2 22.80999947 W
PLW12 0.17502500 W
PLW13 0.08772253 W

F2 - Processing parameters
SI 32768
SF 100.5262565 MHz
WDW EM
SSB 0
LB 1.00 Hz
GB 0
PC 1.40

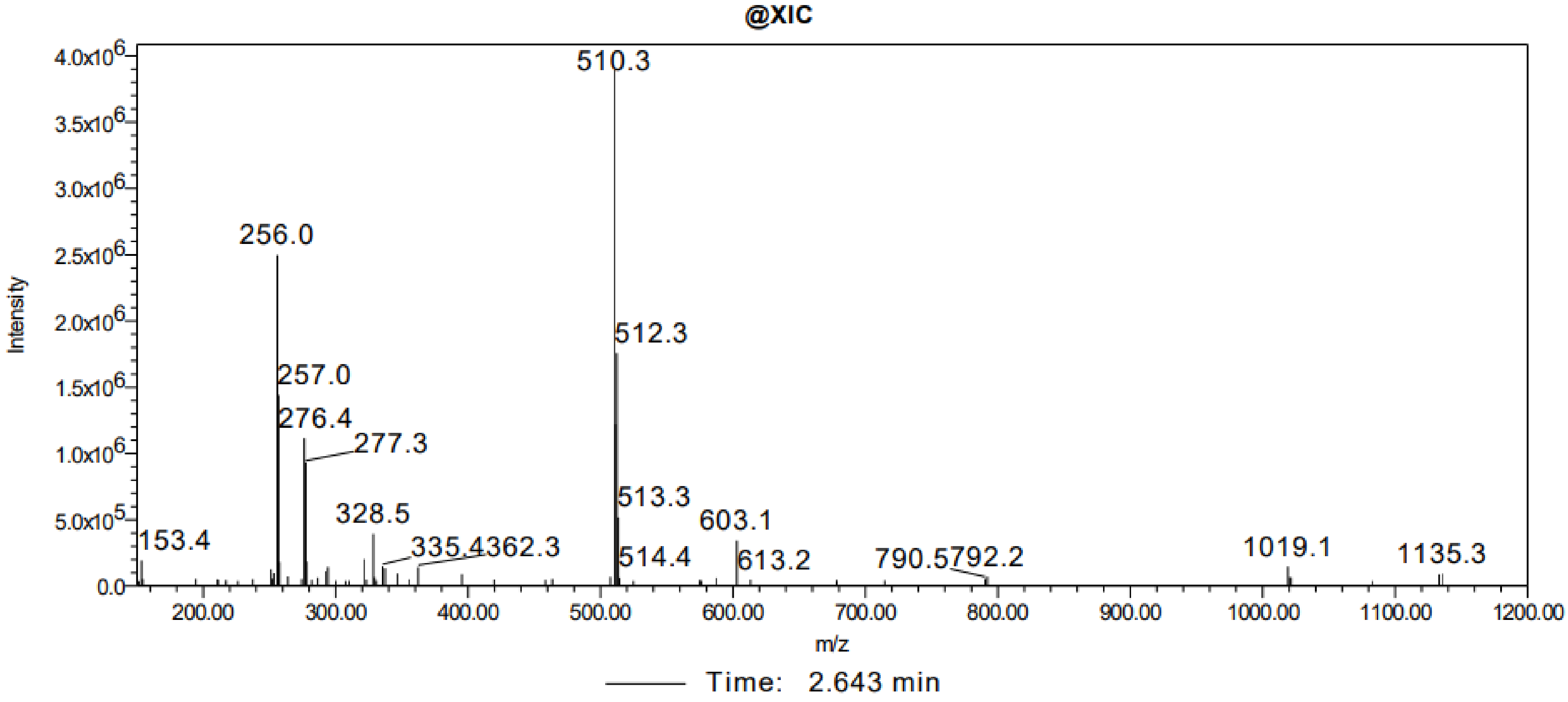

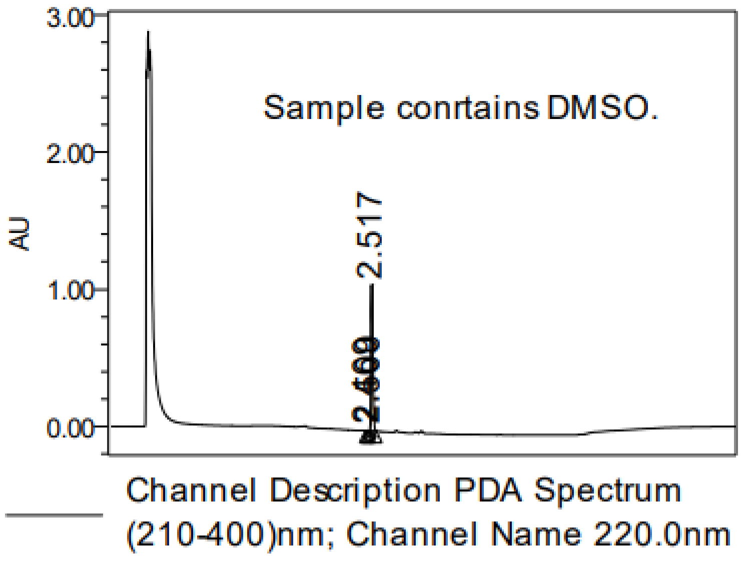

Processed Channel Descr.: PDA 220.0 nm (PDA Spectrum (210-400)nm)

	Processed Channel Descr.	Retention Time (min)	Area	% Area	Purity Angle	Purity Threshold	USP Tailing	USP Plate Count
1	PDA 220.0 nm (PDA Spectrum (210-400)nm)	2.469	1110	0.10	41.34	90.00	1.19	277769
2	PDA 220.0 nm (PDA Spectrum (210-400)nm)	2.500	1627	0.14				
3	PDA 220.0 nm (PDA Spectrum (210-400)nm)	2.517	1128972	99.76	0.18	0.66	1.45	129949

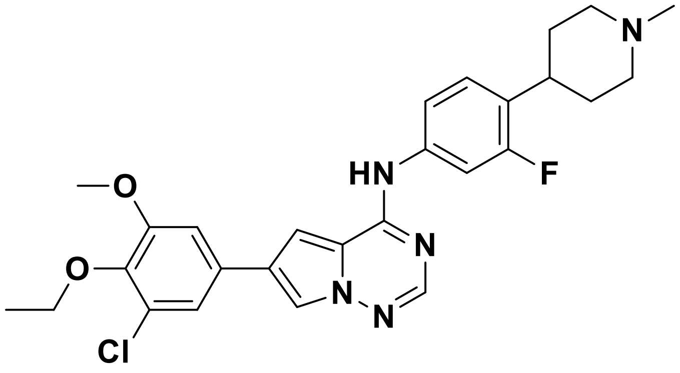

1K2

Chemical Formula: $C_{27}H_{29}ClFN_5O_2$

Exact Mass: 509.1994

Molecular Weight: 510.0104

Sample Name : BDS-007
Test Name : HRMS-1

INDIAN INSTITUTE OF TECHNOLOGY
ROPAR

XEVO G2-XS QTOF

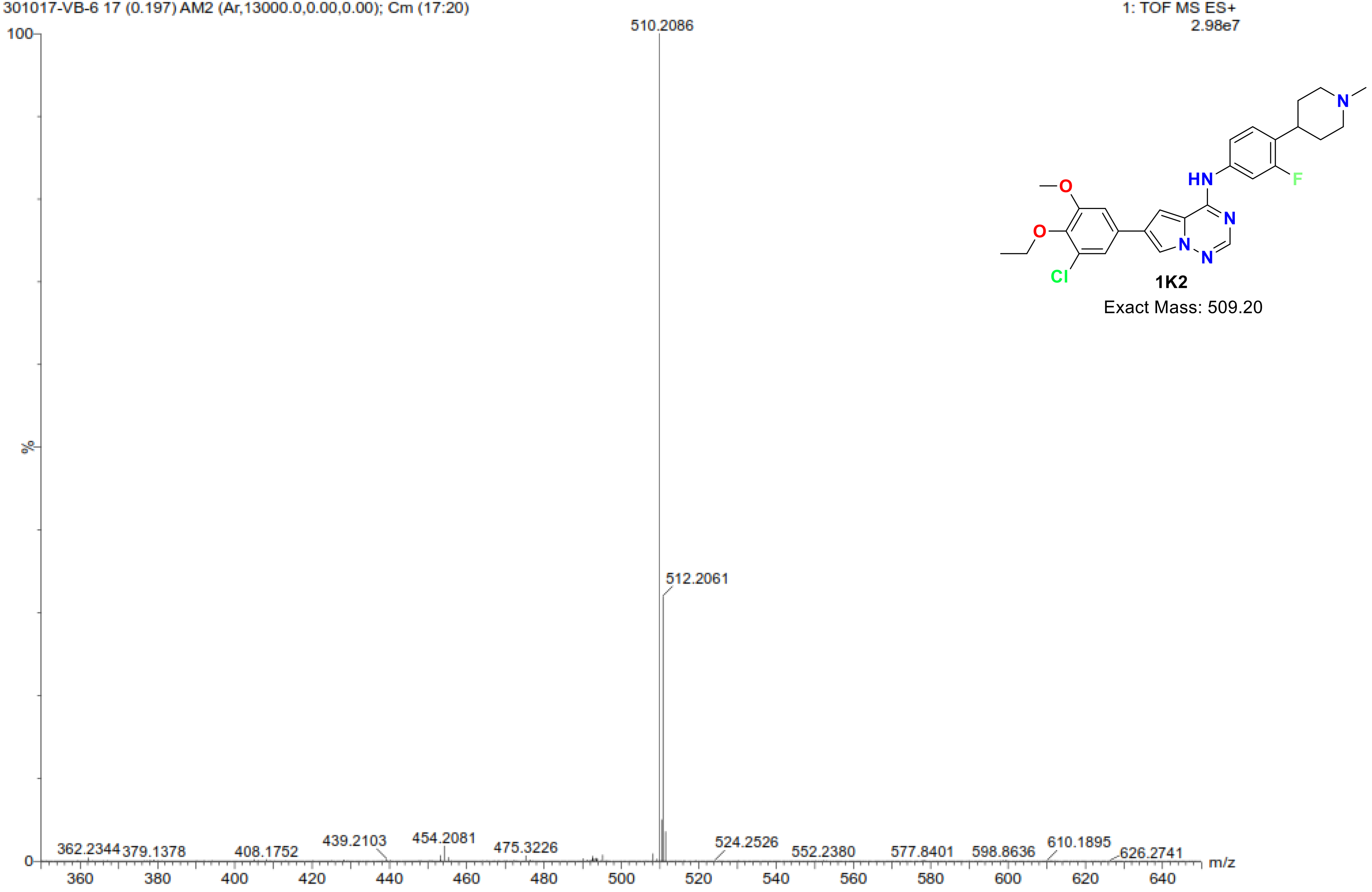

1K2

Exact Mass: 509.20

BS-IBS-023-010-P1

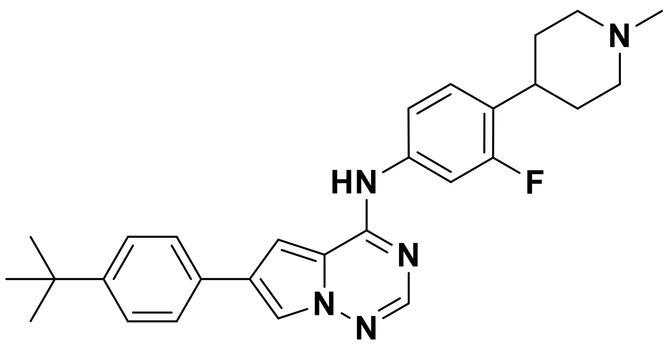

1K3

Chemical Formula: $C_{28}H_{32}FN_5$

Exact Mass: 457.2642

Molecular Weight: 457.5974

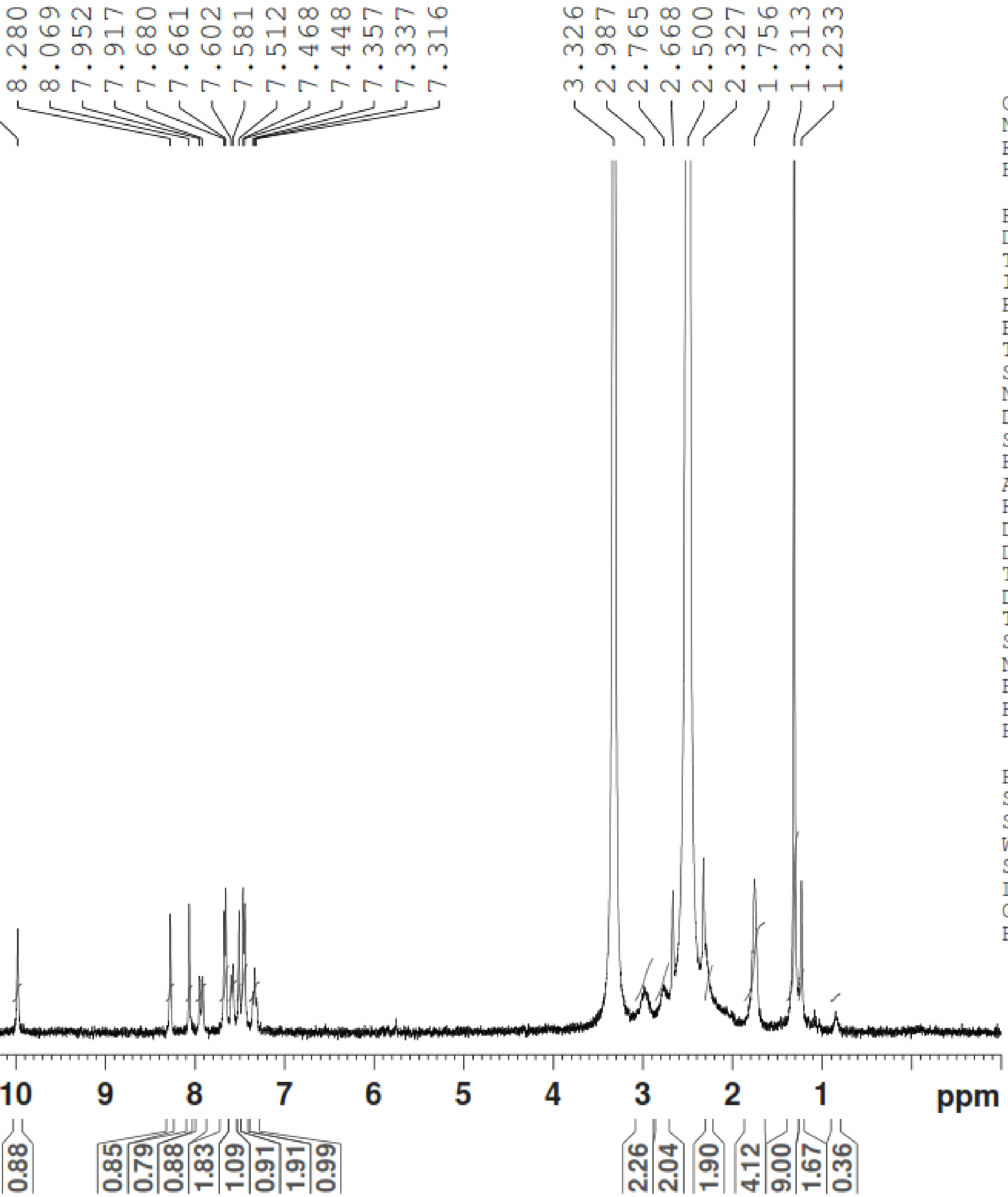

Current Data Parameters
NAME BS-IBS-023-010-P1
EXPNO 17
PROCNO 1

F2 - Acquisition Parameters
Date_ 20230123
Time 11.21 h
INSTRUM AvanceNeo
PROBHD Z163739_0475 (
PULPROG zg30
TD 32768
SOLVENT DMSO
NS 16
DS 0
SWH 7812.500 Hz
FIDRES 0.476837 Hz
AQ 2.0971520 sec
RG 101
DW 64.000 usec
DE 6.64 usec
TE 295.7 K
D1 1.00000000 sec
TD0 1
SFO1 399.7884687 MHz
NUC1 1H
P0 2.67 usec
P1 8.00 usec
PLW1 22.80999947 W

F2 - Processing parameters
SI 16384
SF 399.7860029 MHz
WDW EM
SSB 0
LB 0.30 Hz
GB 0
PC 1.00

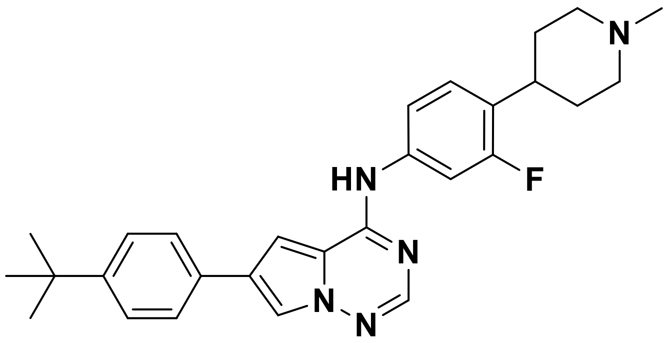

1K3

Chemical Formula: $C_{28}H_{32}FN_5$

Exact Mass: 457.2642

Molecular Weight: 457.5974

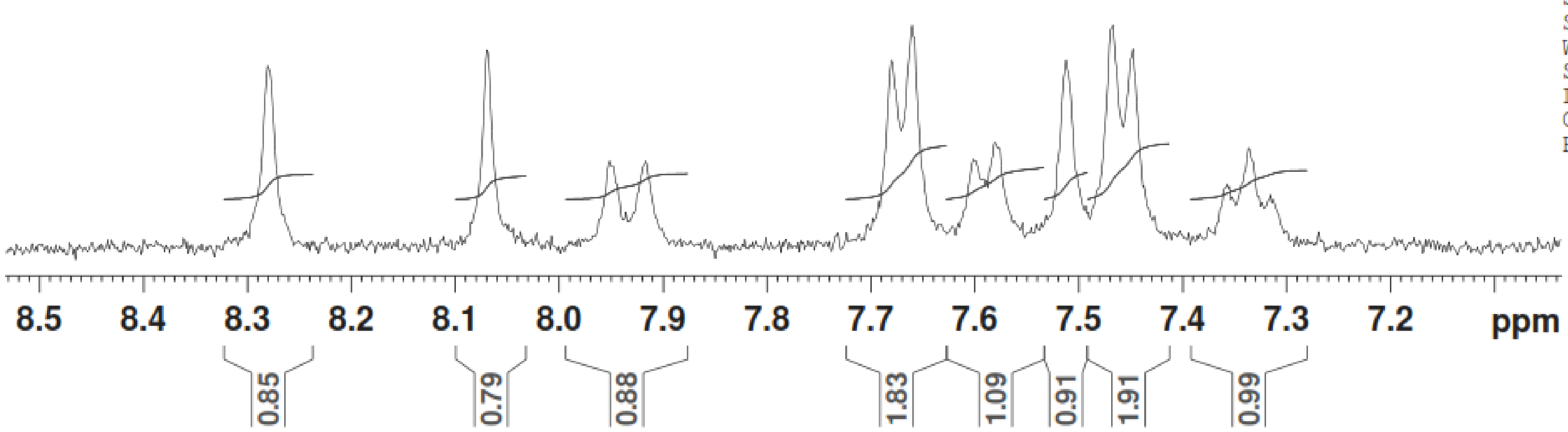


```
Current Data Parameters
NAME      BS-IBS-023-010-P1
EXPNO                    17
PROCNO                    1

F2 - Acquisition Parameters
Date_              20230123
Time                  11.21 h
INSTRUM           AvanceNeo
PROBHD     Z163739_0475 (
PULPROG                zg30
TD                    32768
SOLVENT                DMSO
NS                       16
DS                        0
SWH                7812.500 Hz
FIDRES             0.476837 Hz
AQ                2.0971520 sec
RG                      101
DW                   64.000 usec
DE                     6.64 usec
TE                    295.7 K
D1               1.00000000 sec
TD0                       1
SFO1            399.7884687 MHz
NUC1                     1H
P0                     2.67 usec
P1                     8.00 usec
PLW1            22.80999947 W

F2 - Processing parameters
SI                    16384
SF              399.7860029 MHz
WDW                      EM
SSB                       0
LB                     0.30 Hz
GB                        0
PC                     1.00
```

BS-IBS-023-010-P-C13

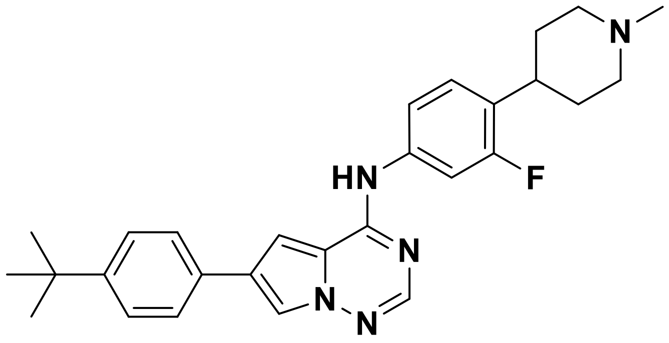

1K3

Chemical Formula: $C_{28}H_{32}FN_5$

Exact Mass: 457.2642

Molecular Weight: 457.5974

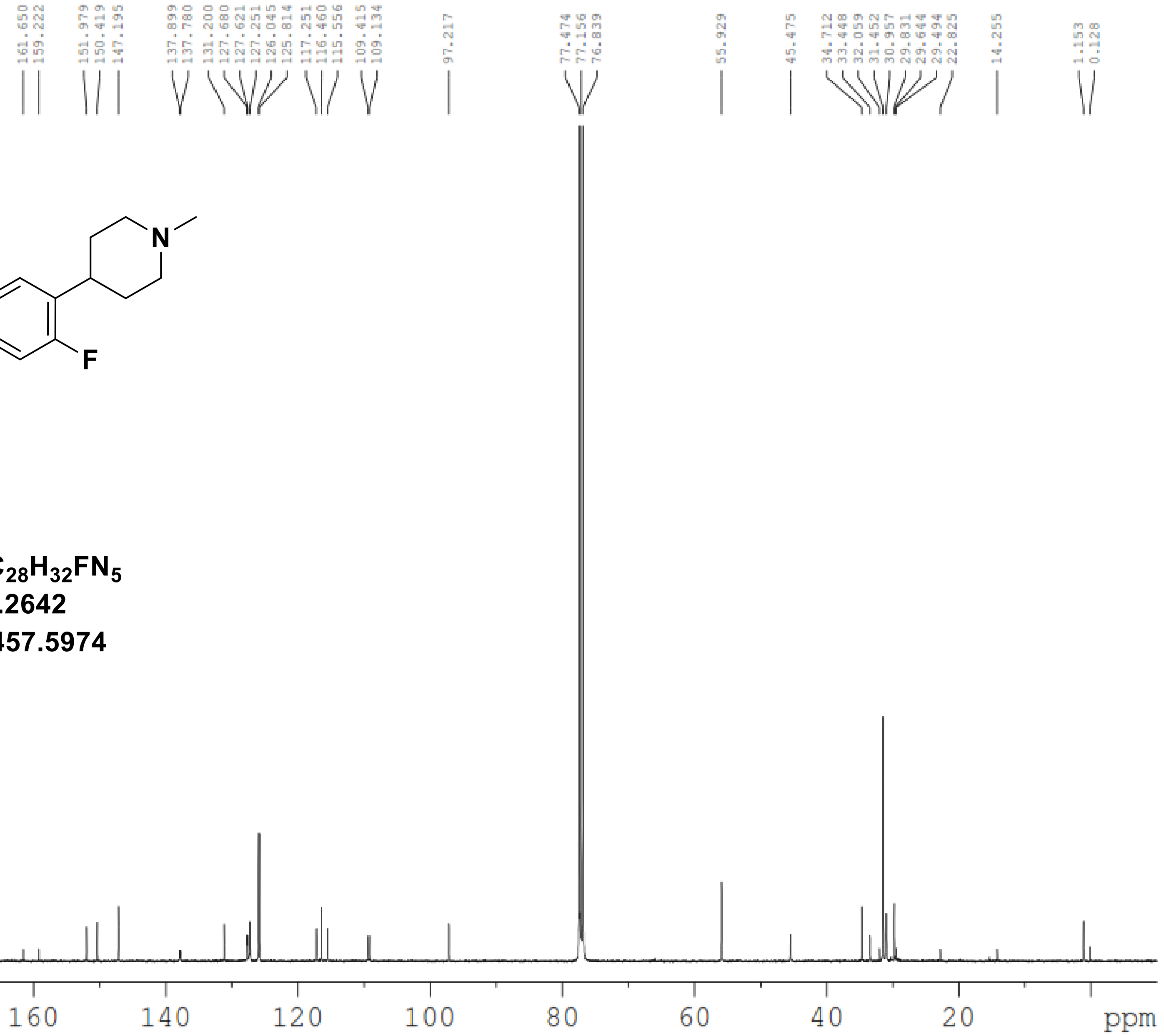

Current Data Parameters

NAME	BS-IBS-023-010-P-C13	
EXPNO	11	
PROCNO	1	

F2 - Acquisition Parameters

Date_	20230124	
Time	7.59	h
INSTRUM	AvanceNeo	
PROBHD	Z163739_0475 (	
PULPROG	zgpg30	
TD	65536	
SOLVENT	CDCl3	
NS	15360	
DS	4	
SWH	23809.523	Hz
FIDRES	0.726609	Hz
AQ	1.3762560	sec
RG	30.1786	
DW	21.000	usec
DE	6.50	usec
TE	297.4	K
D1	2.00000000	sec
D11	0.03000000	sec
TD0	1	
SFO1	100.5363223	MHz
NUC1	13C	
P0	2.67	usec
P1	8.00	usec
PLW1	100.66999817	W
SFO2	399.7875991	MHz
NUC2	1H	
CPDPRG[2	waltz65	
PCPD2	90.00	usec
PLW2	22.80999947	W
PLW12	0.17502500	W
PLW13	0.08772253	W

F2 - Processing parameters

SI	32768	
SF	100.5262573	MHz
WDW	EM	
SSB	0	
LB	1.00	Hz
GB	0	
PC	1.40	

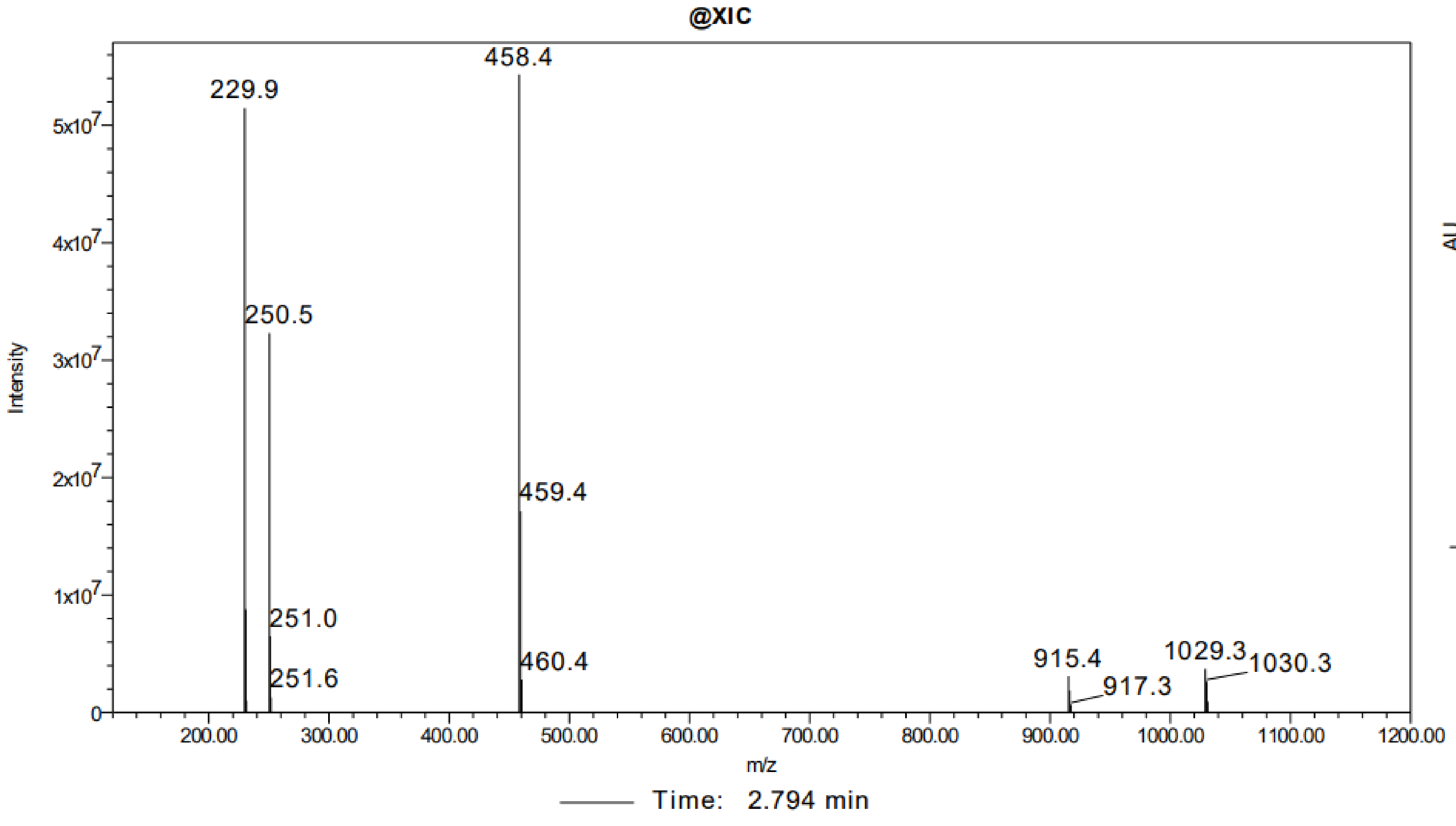

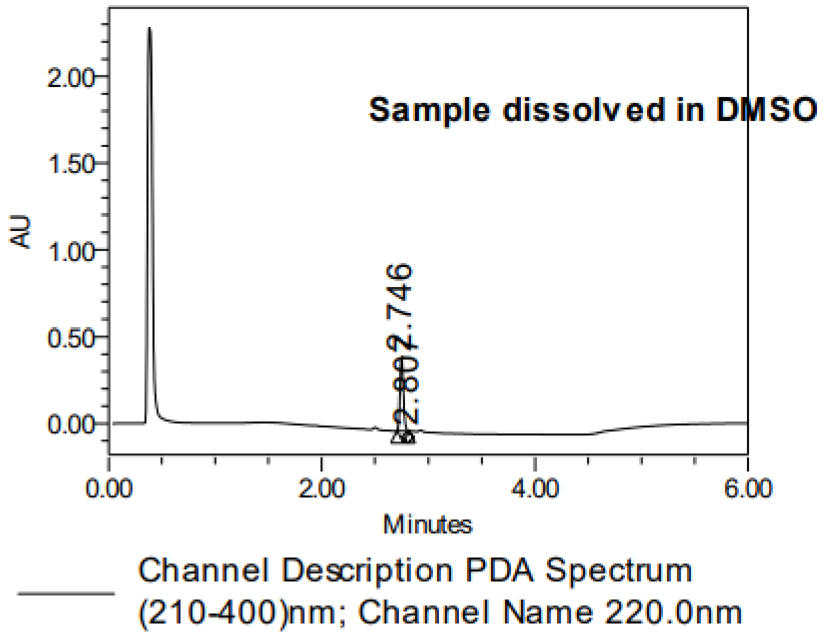

Processed Channel Descr.: PDA 220.0 nm (PDA Spectrum (210-400)nm)

	Processed Channel Descr.	Retention Time (min)	Area	% Area	Purity Angle	Purity Threshold
1	PDA 220.0 nm (PDA Spectrum (210-400)nm)	2.746	925376	99.88	0.08	0.24
2	PDA 220.0 nm (PDA Spectrum (210-400)nm)	2.807	1101	0.12		

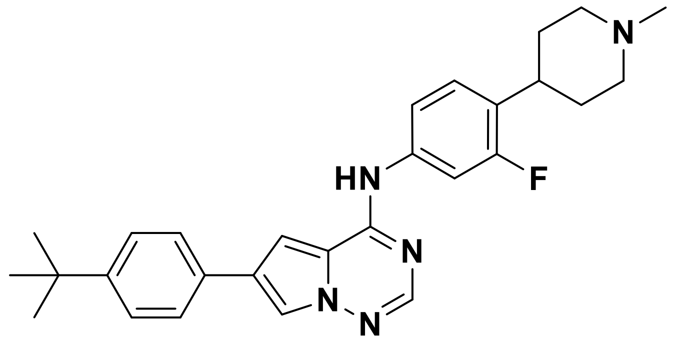

1K3

Chemical Formula: $C_{28}H_{32}FN_5$

Exact Mass: 457.2642

Molecular Weight: 457.5974

Sample Name : BDS-008
Test Name : HRMS-1

INDIAN INSTITUTE OF TECHNOLOGY
ROPAR

XEVO G2-XS QTOF

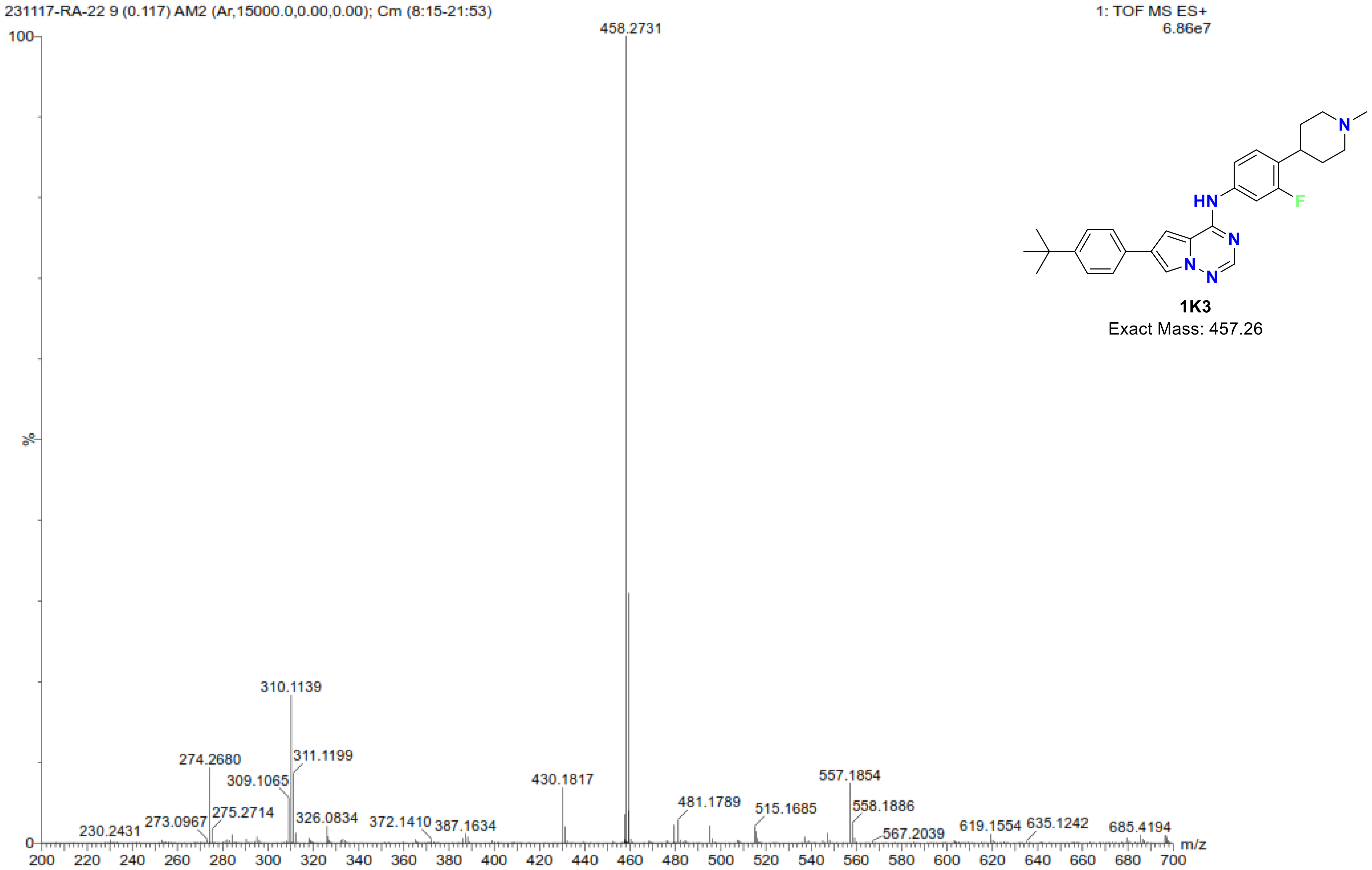

1K3
Exact Mass: 457.26

BS-IBS-023-013-P

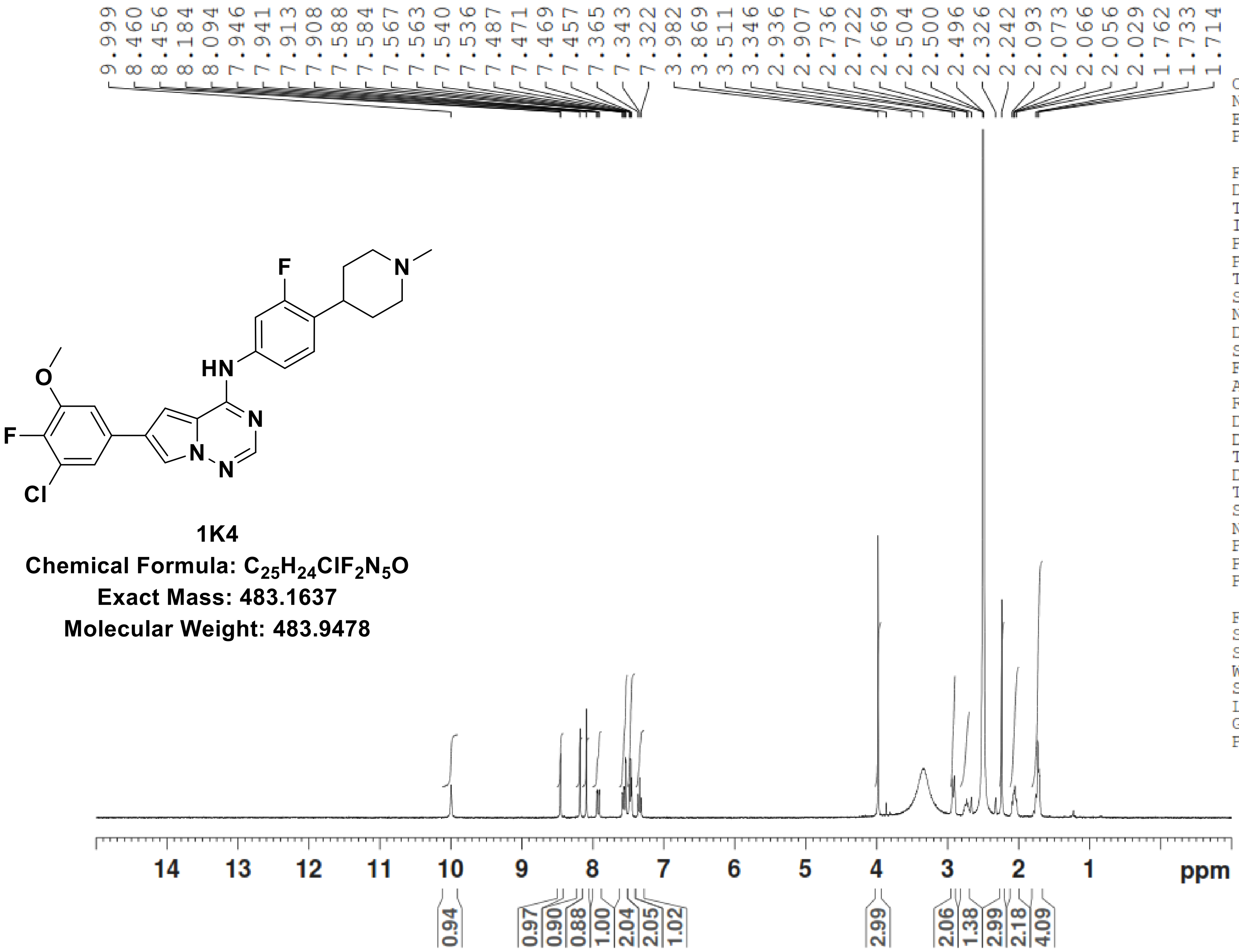

1K4

Chemical Formula: $C_{25}H_{24}ClF_2N_5O$

Exact Mass: 483.1637

Molecular Weight: 483.9478

Current Data Parameters
NAME BS-IBS-023-013-P
EXPNO 27
PROCNO 1

F2 - Acquisition Parameters
Date_ 20230210
Time 17.21 h
INSTRUM AvanceNeo
PROBHD Z163739_0475 (
PULPROG zg30
TD 32768
SOLVENT DMSO
NS 8
DS 0
SWH 7812.500 Hz
FIDRES 0.476837 Hz
AQ 2.0971520 sec
RG 101
DW 64.000 usec
DE 6.64 usec
TE 296.9 K
D1 1.00000000 sec
TD0 1
SFO1 399.7884687 MHz
NUC1 1H
P0 2.67 usec
P1 8.00 usec
PLW1 22.80999947 W

F2 - Processing parameters
SI 16384
SF 399.7860029 MHz
WDW EM
SSB 0
LB 0.30 Hz
GB 0
PC 1.00

BS-IBS-023-013-P

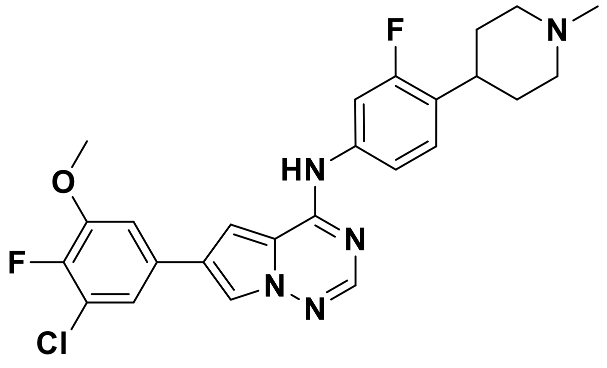

1K4
Chemical Formula: $C_{25}H_{24}ClF_2N_5O$
Exact Mass: 483.1637
Molecular Weight: 483.9478

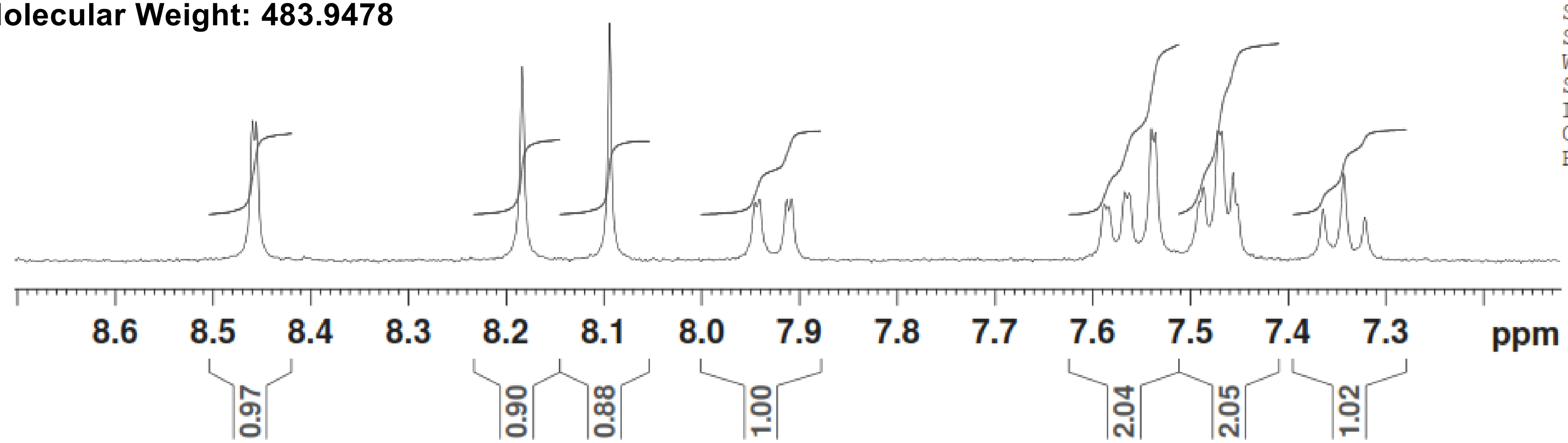

Current Data Parameters
NAME BS-IBS-023-013-P
EXPNO 27
PROCNO 1

F2 - Acquisition Parameters
Date_ 20230210
Time 17.21 h
INSTRUM AvanceNeo
PROBHD Z163739_0475 (
PULPROG zg30
TD 32768
SOLVENT DMSO
NS 8
DS 0
SWH 7812.500 Hz
FIDRES 0.476837 Hz
AQ 2.0971520 sec
RG 101
DW 64.000 usec
DE 6.64 usec
TE 296.9 K
D1 1.00000000 sec
TD0 1
SFO1 399.7884687 MHz
NUC1 1H
P0 2.67 usec
P1 8.00 usec
PLW1 22.80999947 W

F2 - Processing parameters
SI 16384
SF 399.7860029 MHz
WDW EM
SSB 0
LB 0.30 Hz
GB 0
PC 1.00

BS-IBS-023-013-13C

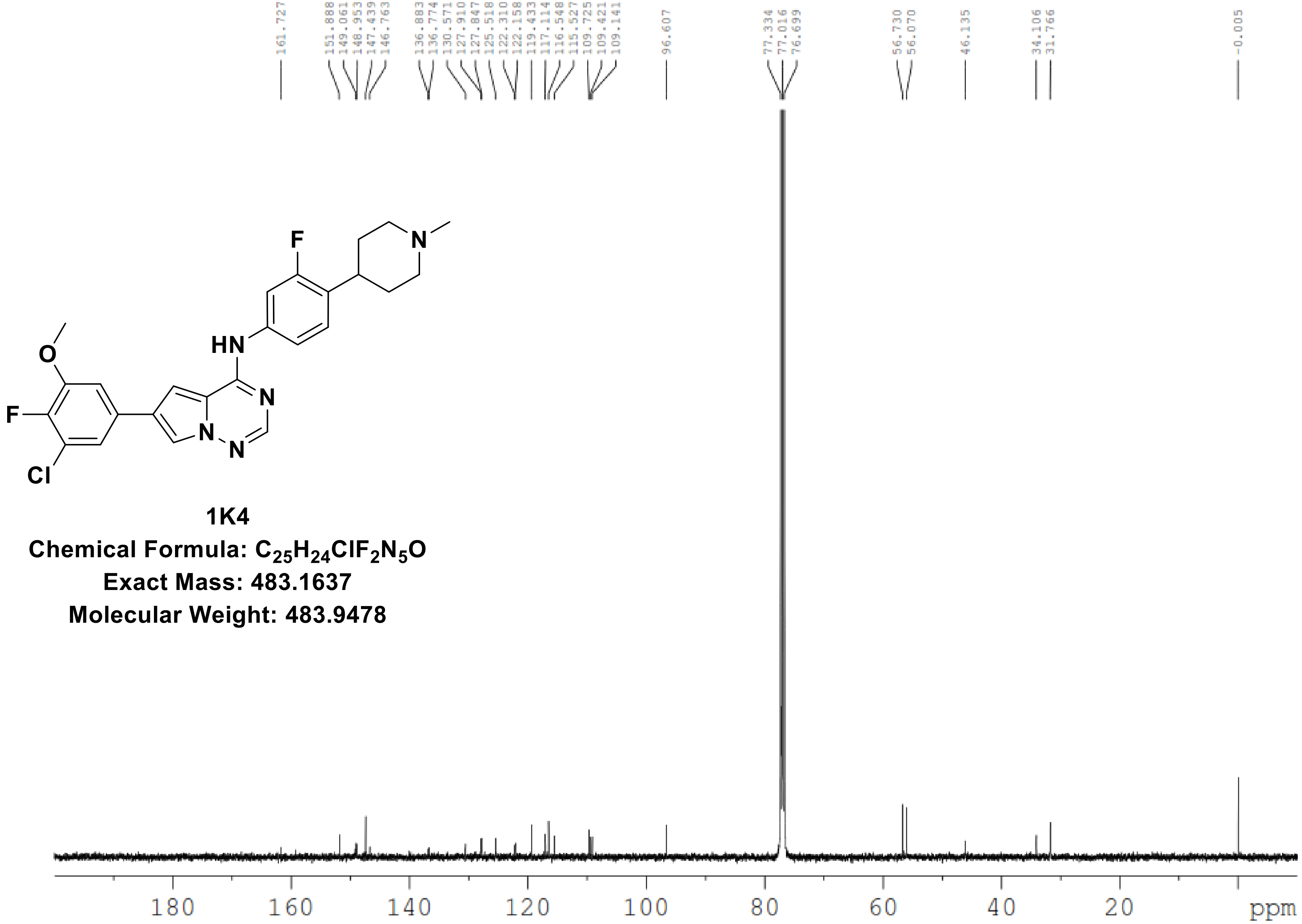

1K4

Chemical Formula: $C_{25}H_{24}ClF_2N_5O$

Exact Mass: 483.1637

Molecular Weight: 483.9478

Current Data Parameters

NAME	BS-IBS-023-013-13C
EXPNO	45
PROCNO	1

F2 - Acquisition Parameters

Date_	20230502
Time	8.24 h
INSTRUM	AvanceNeo
PROBHD	Z163739_0475 (
PULPROG	zgpg30
TD	65536
SOLVENT	CDCl3
NS	15360
DS	4
SWH	23809.523 Hz
FIDRES	0.726609 Hz
AQ	1.3762560 sec
RG	101
DW	21.000 usec
DE	6.50 usec
TE	298.6 K
D1	2.00000000 sec
D11	0.03000000 sec
TD0	1
SFO1	100.5363223 MHz
NUC1	13C
P0	2.67 usec
P1	8.00 usec
PLW1	100.66999817 W
SFO2	399.7875991 MHz
NUC2	1H
CPDPRG[2	waltz65
PCPD2	90.00 usec
PLW2	22.80999947 W
PLW12	0.17502500 W
PLW13	0.08772253 W

F2 - Processing parameters

SI	32768
SF	100.5262697 MHz
WDW	EM
SSB	0
LB	1.00 Hz
GB	0
PC	1.40

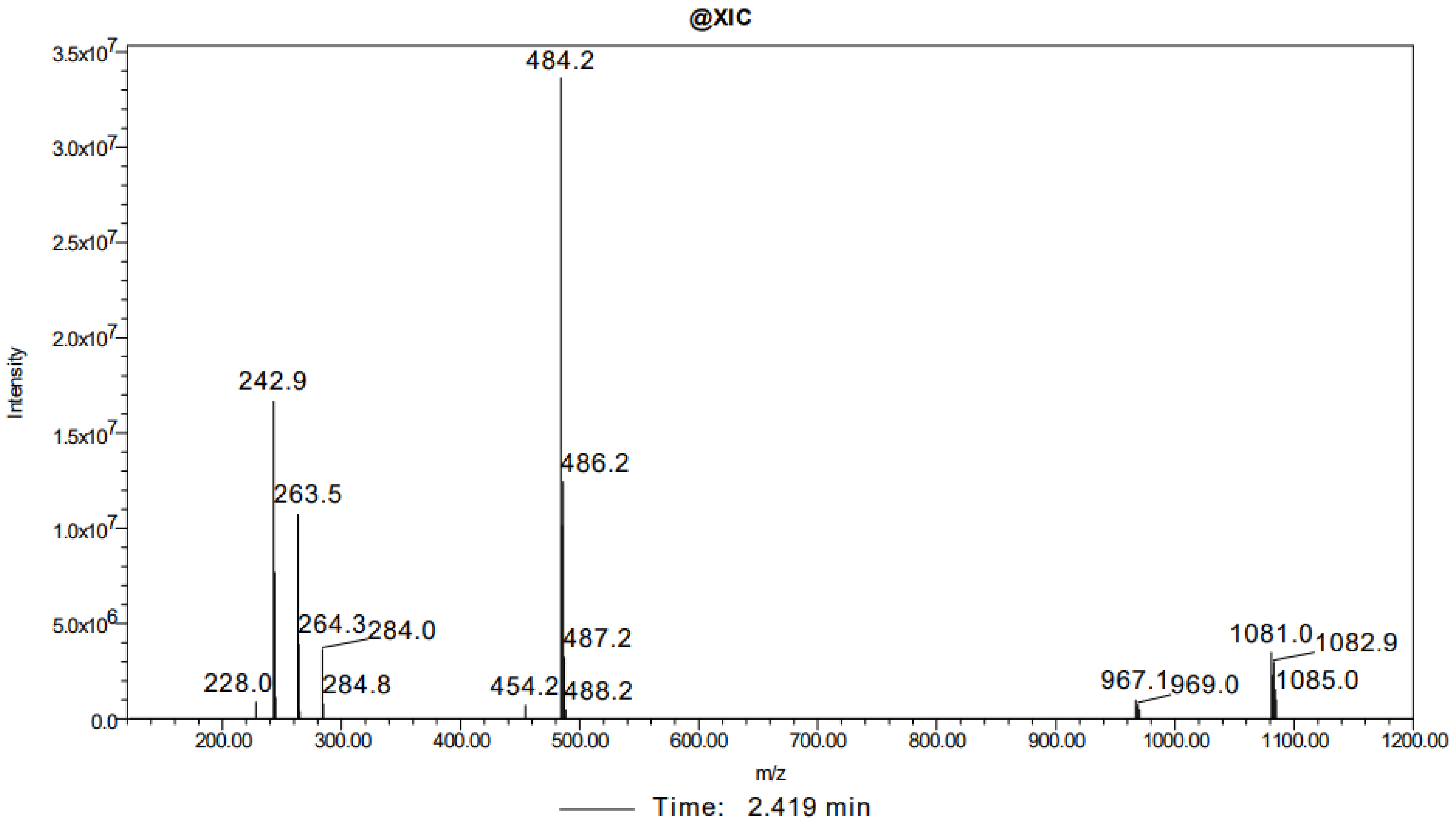

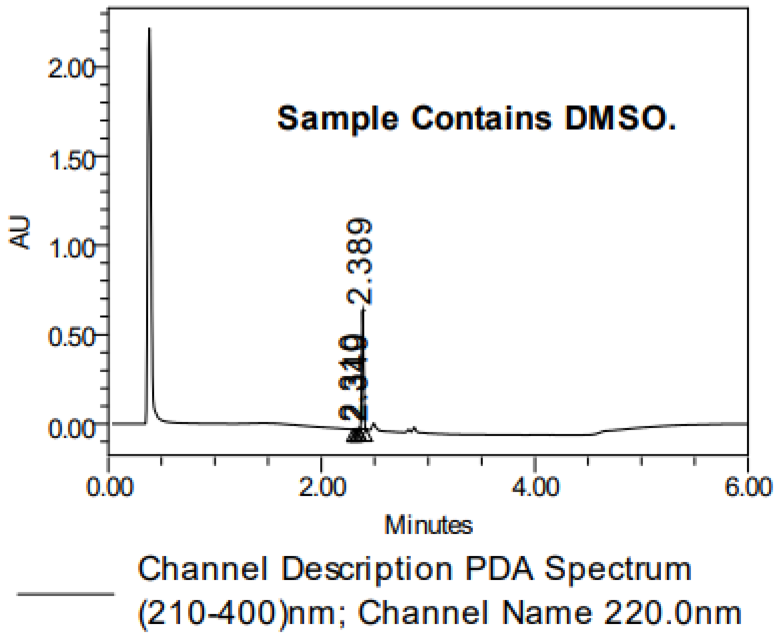

Processed Channel Descr.: PDA 220.0 nm (PDA Spectrum (210-400)nm)

	Processed Channel Descr.	Retention Time (min)	Area	% Area	Purity Angle	Purity Threshold
1	PDA 220.0 nm (PDA Spectrum (210-400)nm)	2.310	1969	0.23		
2	PDA 220.0 nm (PDA Spectrum (210-400)nm)	2.349	3689	0.43		
3	PDA 220.0 nm (PDA Spectrum (210-400)nm)	2.389	846930	99.34	0.17	0.25

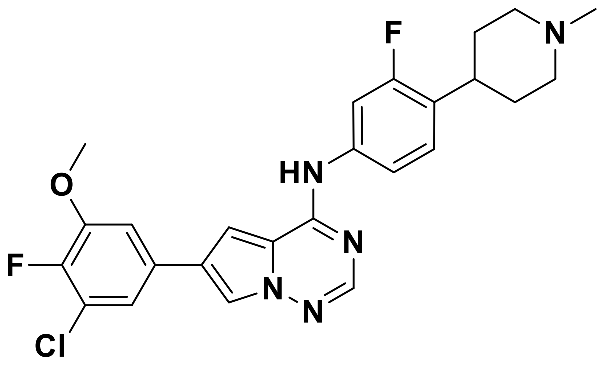

1K4

Chemical Formula: $C_{25}H_{24}ClF_2N_5O$

Exact Mass: 483.1637

Molecular Weight: 483.9478

Sample Name : BDS-009
Test Name : HRMS-1

INDIAN INSTITUTE OF TECHNOLOGY
ROPAR

XEVO G2-XS QTOF

231117-SS-08 16 (0.177) AM2 (Ar,15000.0,0.00,0.00); Cm (16:17)

1: TOF MS ES+
9.38e6

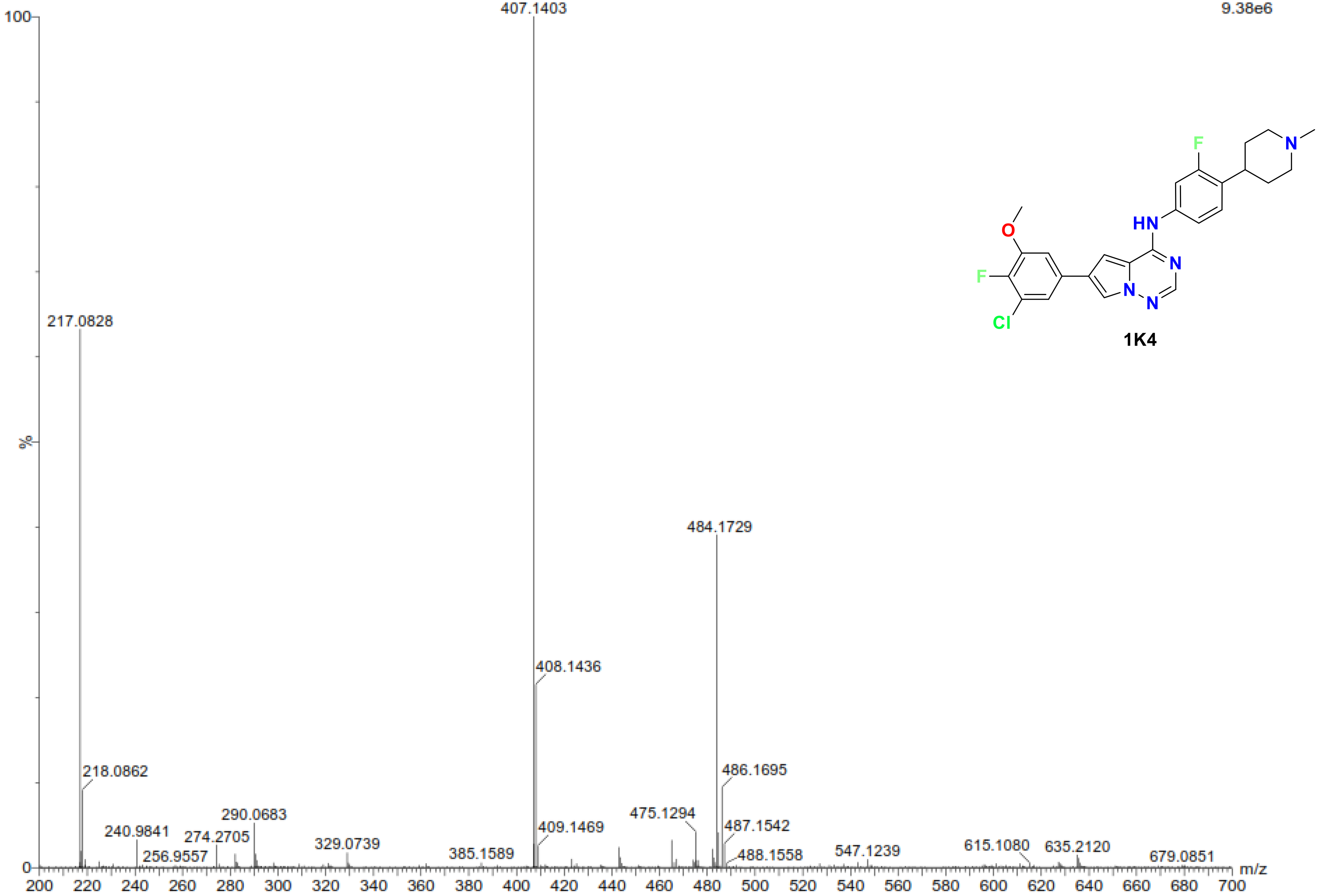

1K4

BS-IBS-023-011-F

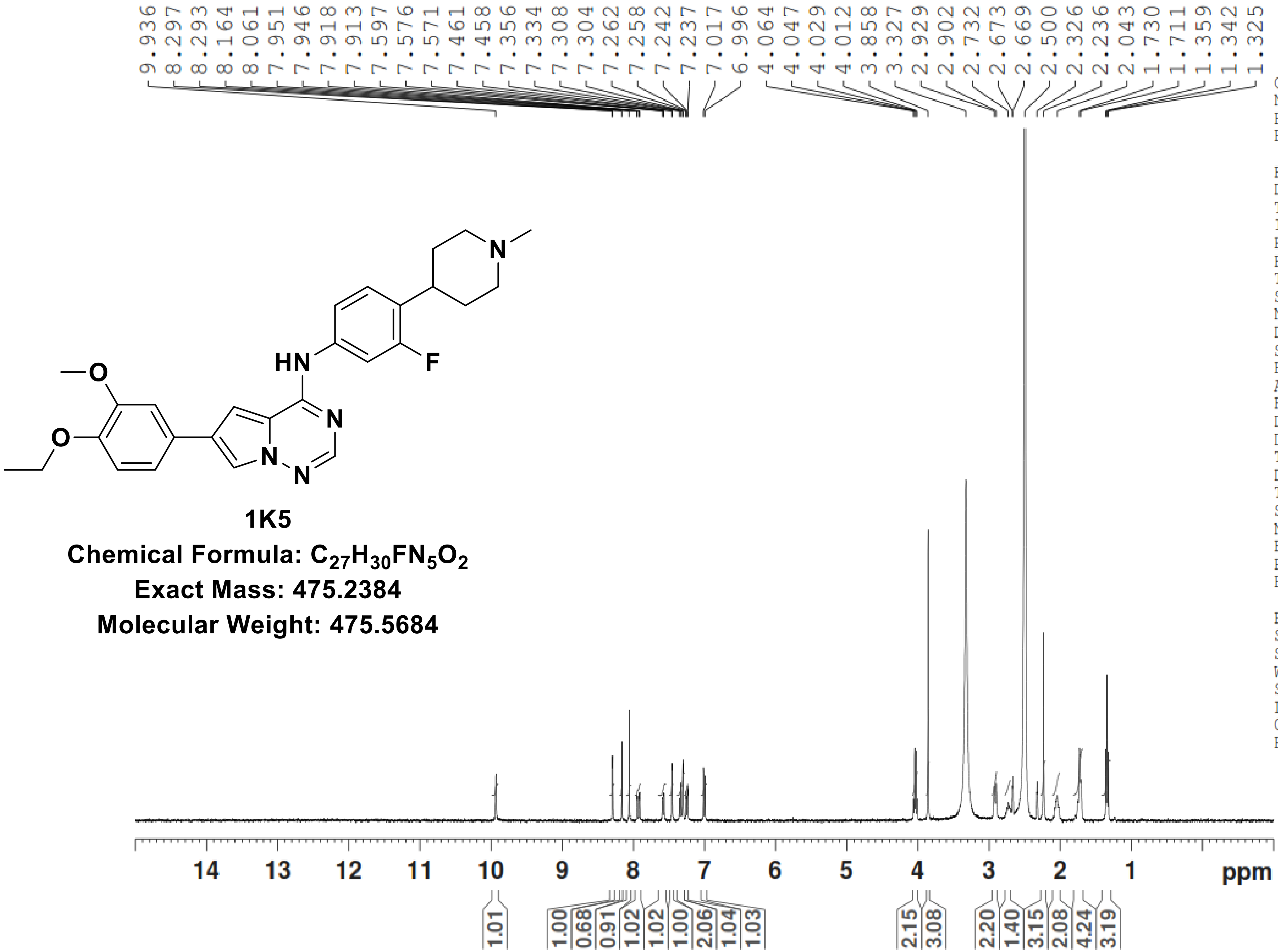

1K5
Chemical Formula: $C_{27}H_{30}FN_5O_2$
Exact Mass: 475.2384
Molecular Weight: 475.5684

Current Data Parameters
NAME BS-IBS-023-011-F
EXPNO 40
PROCNO 1

F2 - Acquisition Parameters
Date_ 20230223
Time 11.10 h
INSTRUM AvanceNeo
PROBHD Z163739_0475 (
PULPROG zg30
TD 32768
SOLVENT DMSO
NS 8
DS 0
SWH 7812.500 Hz
FIDRES 0.476837 Hz
AQ 2.0971520 sec
RG 101
DW 64.000 usec
DE 6.64 usec
TE 295.4 K
D1 1.00000000 sec
TD0 1
SFO1 399.7884687 MHz
NUC1 1H
P0 2.67 usec
P1 8.00 usec
PLW1 22.80999947 W

F2 - Processing parameters
SI 16384
SF 399.7860029 MHz
WDW EM
SSB 0
LB 0.30 Hz
GB 0
PC 1.00

BS-IBS-023-011-F

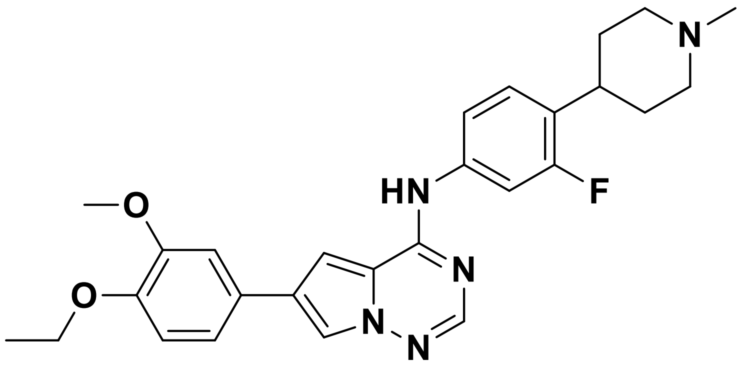

1K5

Chemical Formula: $C_{27}H_{30}FN_5O_2$

Exact Mass: 475.2384

Molecular Weight: 475.5684

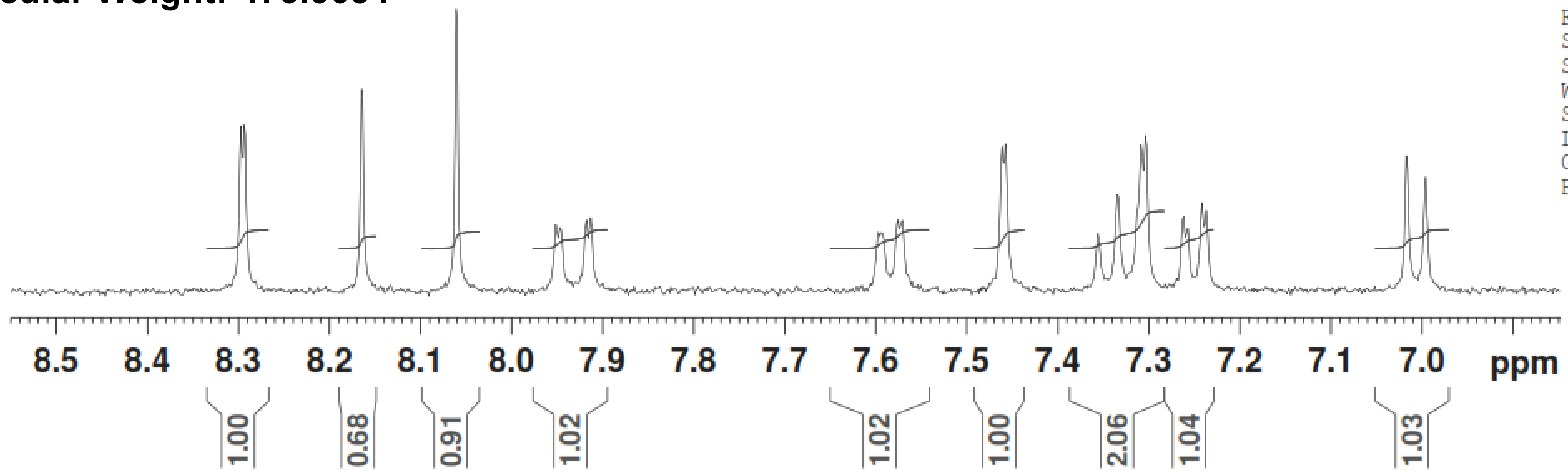

Current Data Parameters

NAME	BS-IBS-023-011-F
EXPNO	40
PROCNO	1

F2 - Acquisition Parameters

Date_	20230223
Time	11.10 h
INSTRUM	AvanceNeo
PROBHD	Z163739_0475 (
PULPROG	zg30
TD	32768
SOLVENT	DMSO
NS	8
DS	0
SWH	7812.500 Hz
FIDRES	0.476837 Hz
AQ	2.0971520 sec
RG	101
DW	64.000 usec
DE	6.64 usec
TE	295.4 K
D1	1.00000000 sec
TD0	1
SFO1	399.7884687 MHz
NUC1	1H
P0	2.67 usec
P1	8.00 usec
PLW1	22.80999947 W

F2 - Processing parameters

SI	16384
SF	399.7860029 MHz
WDW	EM
SSB	0
LB	0.30 Hz
GB	0
PC	1.00

BS-IBS-023-011-F-13C

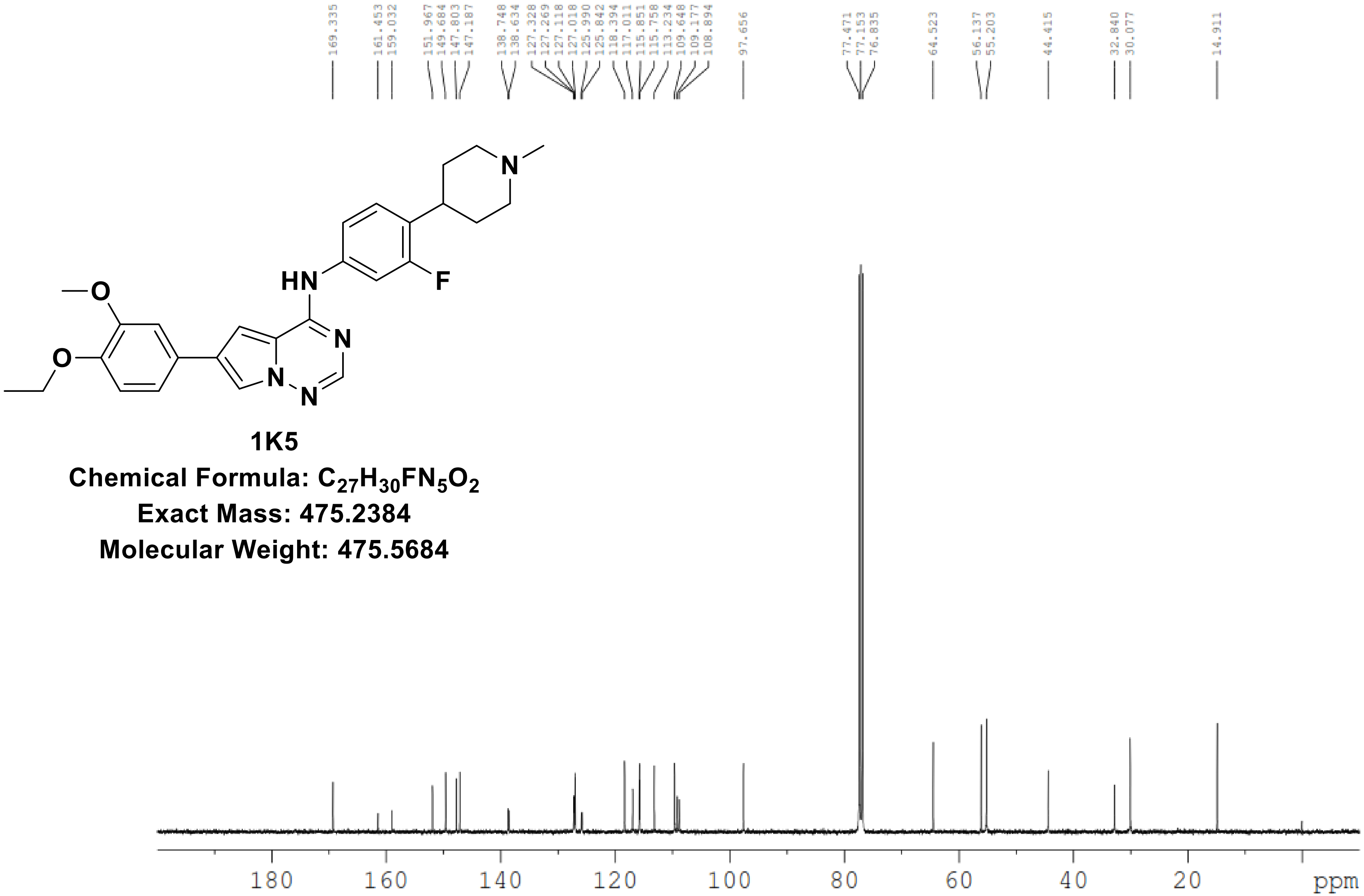

1K5

Chemical Formula: $C_{27}H_{30}FN_5O_2$

Exact Mass: 475.2384

Molecular Weight: 475.5684

Current Data Parameters
NAME BS-IBS-023-011-F-13C
EXPNO 51
PROCNO 1

F2 - Acquisition Parameters
Date_ 20230227
Time 15.30 h
INSTRUM AvanceNeo
PROBHD Z163739_0475 (
PULPROG zgpg30
TD 65536
SOLVENT CDCl3
NS 2048
DS 4
SWH 23809.523 Hz
FIDRES 0.726609 Hz
AQ 1.3762560 sec
RG 42.9639
DW 21.000 usec
DE 6.50 usec
TE 296.9 K
D1 2.00000000 sec
D11 0.03000000 sec
TD0 1
SFO1 100.5363223 MHz
NUC1 13C
P0 2.67 usec
P1 8.00 usec
PLW1 100.66999817 W
SFO2 399.7875991 MHz
NUC2 1H
CPDPRG[2 waltz65
PCPD2 90.00 usec
PLW2 22.80999947 W
PLW12 0.17502500 W
PLW13 0.08772253 W

F2 - Processing parameters
SI 32768
SF 100.5262608 MHz
WDW EM
SSB 0
LB 1.00 Hz
GB 0
PC 1.40

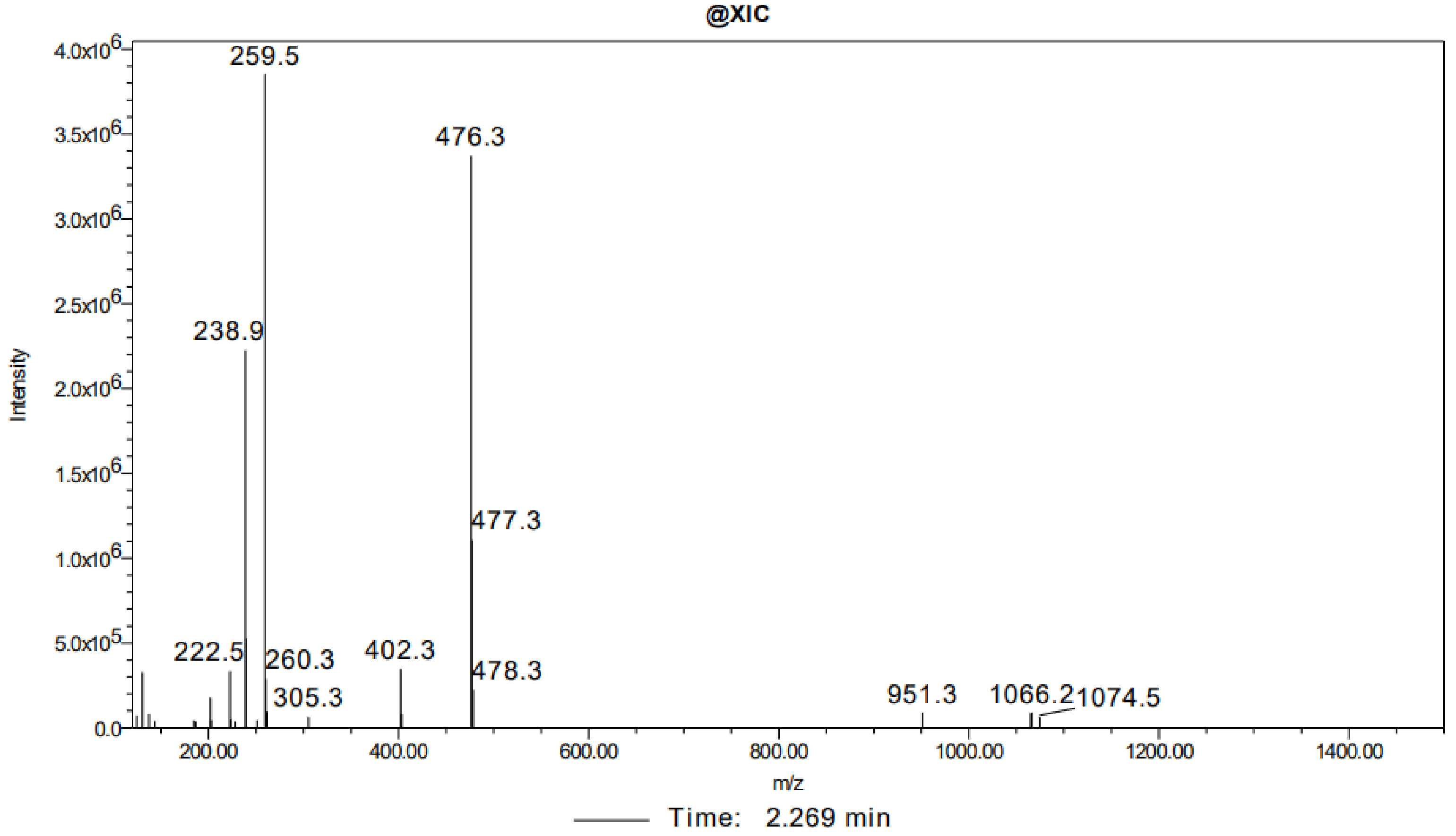

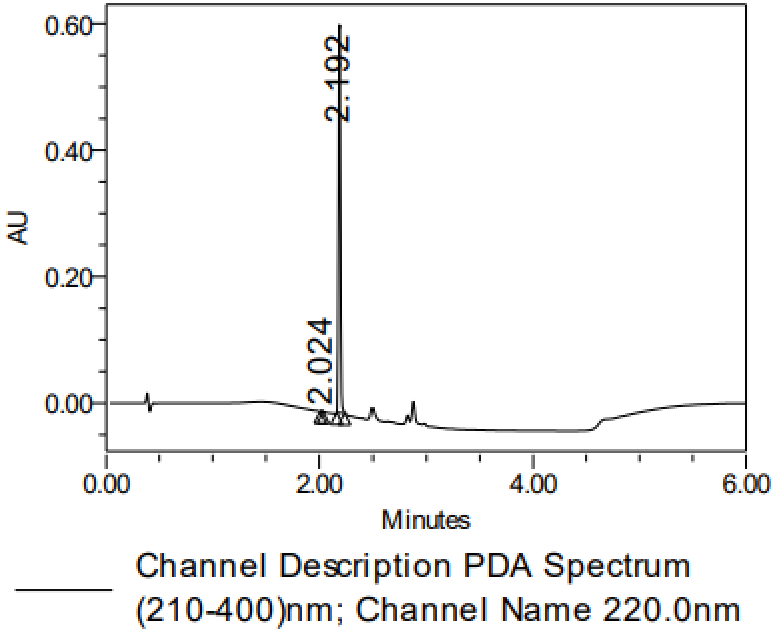

Processed Channel Descr.: PDA 220.0 nm (PDA Spectrum (210-400)nm)

	Processed Channel Descr.	Retention Time (min)	Area	% Area	Purity Angle	Purity Threshold	USP Tailing	USP Plate Count
1	PDA 220.0 nm (PDA Spectrum (210-400)nm)	2.024	1212	0.15			1.09	75760
2	PDA 220.0 nm (PDA Spectrum (210-400)nm)	2.192	794251	99.85	0.17	0.25	1.05	62026

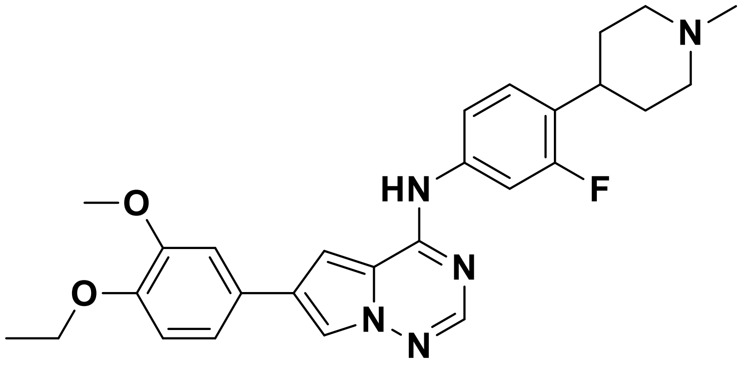

1K5

Chemical Formula: $C_{27}H_{30}FN_5O_2$

Exact Mass: 475.2384

Molecular Weight: 475.5684

Sample Name : BDS-010
Test Name : HRMS-1

INDIAN INSTITUTE OF TECHNOLOGY
ROPAR

XEVO G2-XS QTOF

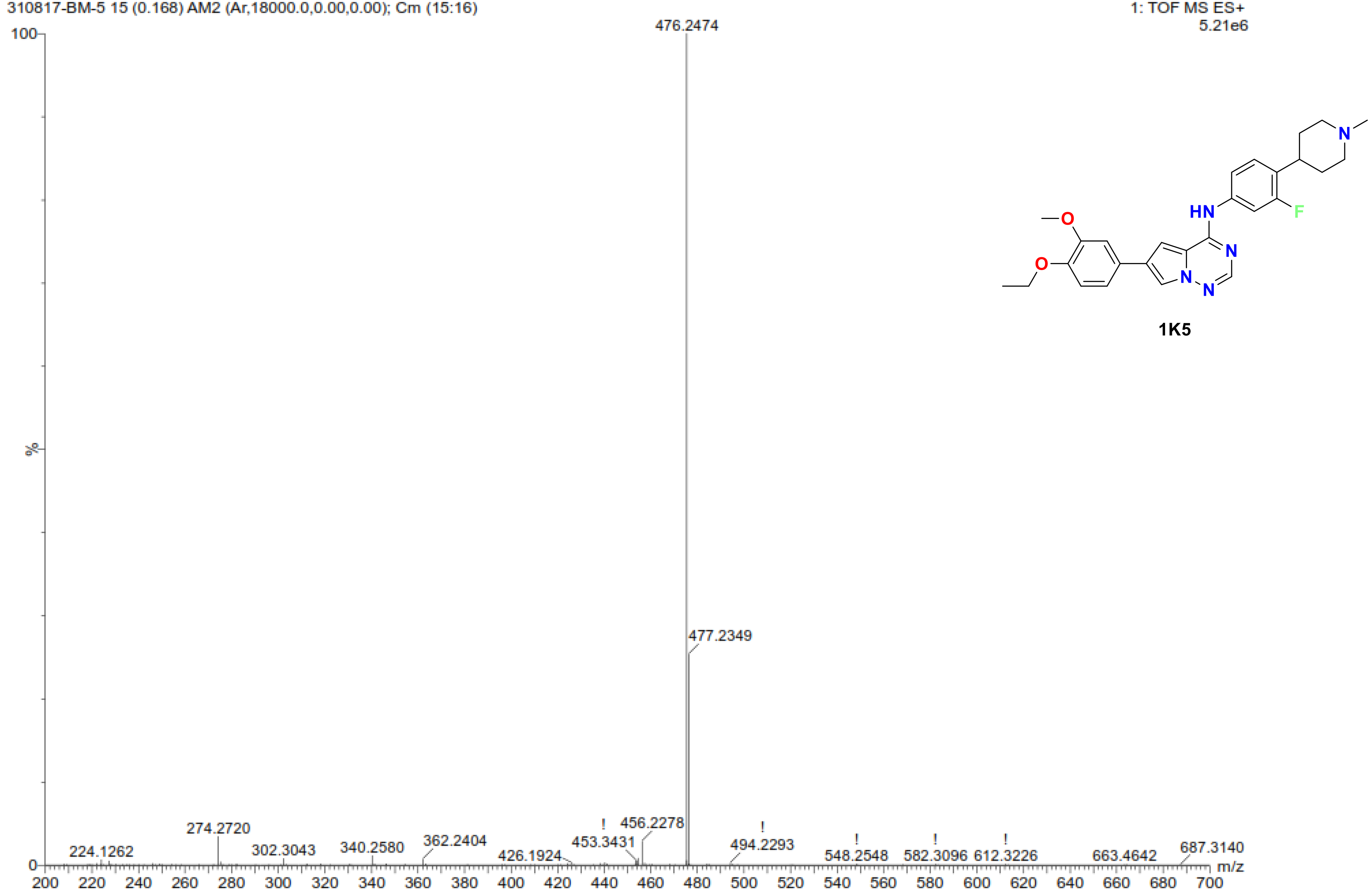

1K5
